# *PgLOX6* encoding a lipoxygenase contributes to jasmonic acid biosynthesis and ginsenoside production in *Panax ginseng*

**DOI:** 10.1093/jxb/erw358

**Published:** 2016-10-06

**Authors:** Shadi Rahimi, Yu-Jin Kim, Johan Sukweenadhi, Dabing Zhang, Deok-Chun Yang

**Affiliations:** ^1^Graduate School of Biotechnology and Ginseng Bank, College of Life Sciences, Kyung Hee University, Yongin, 446–701, Republic of Korea; ^2^Department of Crop Science, Chungbuk National University, Cheongju 361–763, Korea; ^3^Department of Oriental Medicinal Biotechnology, College of Life Sciences, Kyung Hee University, Yongin, 446–701, Republic of Korea; ^4^State Key Laboratory of Hybrid Rice, Shanghai Jiao Tong University–University of Adelaide Joint Centre for Agriculture and Health, School of Life Sciences and Biotechnology, Shanghai Jiao Tong University, Shanghai 200240, China; ^5^School of Agriculture, Food and Wine, University of Adelaide, Waite Campus, Urrbrae, South Australia 5064, Australia

**Keywords:** Ginsenoside, jasmonic acid, lipoxygenase, *Panax ginseng*, squalene, secondary metabolite, triterpene, vascular bundles.

## Abstract

Ginsenosides, the valuable pharmaceutical compounds in *Panax ginseng*, are triterpene saponins that occur mainly in ginseng plants. It was shown that *in vitro* treatment with the phytohormone jasmonic acid (JA) is able to increase ginsenoside production in ginseng plants. To understand the molecular link between JA biosynthesis and ginsenoside biosynthesis, we identified a JA biosynthetic 13-lipoxygenase gene (*PgLOX6*) in *P. ginseng* that promotes ginsenoside production. The expression of *PgLOX6* was high in vascular bundles, which corresponds with expression of ginsenoside biosynthetic genes. Consistent with the role of *PgLOX6* in synthesizing JA and promoting ginsenoside synthesis, transgenic plants overexpressing *PgLOX6* in Arabidopsis had increased amounts of JA and methyl jasmonate (MJ), increased expression of triterpene biosynthetic genes such as *squalene synthase* (*AtSS1*) and *squalene epoxidase* (*AtSE1*), and increased squalene content. Moreover, transgenic ginseng roots overexpressing *PgLOX6* had around 1.4-fold increased ginsenoside content and upregulation of ginsenoside biosynthesis-related genes including *PgSS1*, *PgSE1*, and *dammarenediol synthase* (*PgDDS*), which is similar to that of treatment with MJ. However, MJ treatment of transgenic ginseng significantly enhanced JA and MJ, associated with a 2.8-fold increase of ginsenoside content compared with the non-treated, non-transgenic control plant, which was 1.4 times higher than the MJ treatment effect on non-transgenic plants. These results demonstrate that *PgLOX6* is responsible for the biosynthesis of JA and promotion of the production of triterpenoid saponin through up-regulating the expression of ginsenoside biosynthetic genes. This work provides insight into the role of JA in biosynthesizing secondary metabolites and provides a molecular tool for increasing ginsenoside production.

## Highlight

In ginseng, jasmonic acid promotes expression of the biosynthetic genes for ginsenosides. *PgLOX6* encodes a lipoxygenase that is required for biosynthesis of jasmonic acid and its overexpression increases ginsenoside levels.

## Introduction

*Panax* belongs to the family Araliaceae and contains at least 17 species (Kim *et al.*, 2015). Since ancient time, *Panax ginseng* (known as Korean or Asian ginseng) has been considered as a healing drug and health tonic in China, Japan, and other Asian countries (Radad *et al.*, 2006). The pharmacological effects of ginseng are correlated with ginsenosides, bioactive ingredients with a glycosylated triterpene structure. There are more than 150 naturally occurring ginsenosides in *Panax* species, which are classified according to their structure into two types, protopanaxadiols (PPDs) such as ginsenoside Rb1, Rb2, Rb3, Rc, and Rd, and protopanaxatriols (PPTs) such as ginsenoside Re, Rf, Rg1, Rg2, Rh1, and F1. Triterpene ginsenosides are mostly biosynthesized through the mevalonate (MVA) pathway in the cytosol. The first rate limiting reaction of this pathway is catalysed by 3-hydroxy-3-methylglutaryl coenzyme A reductase (HMGR). Subsequently, head-to-head assembly of two farnesyl diphosphate (FPP) molecules produces a C30 molecule, squalene, which is converted to (*S*)-2,3-oxidosqualene (a common precursor of sterols) by the action of squalene synthase (SS) and squalene epoxidase (SE). Then (*S*)-2,3-oxidosqualene undergoes additional cyclization, hydroxylation, and glycosylation by the enzymes dammarenediol synthase (DDS), cytochrome P450 and glycosyltransferase (GT), respectively (Kim *et al.*, 2015; Rahimi *et al.*, 2015*a*).

There are several reports showing that ginsenoside accumulation in cell suspension, adventitious roots, and hairy root ginseng cultures is elicited by jasmonates (JAs) (Rahimi *et al.*, 2015*a*,*b*). Exogenous application of JAs could induce endogenous JA biosynthesis (Hu and Zhong, 2007, 2008) followed by expression of ginsenoside biosynthetic genes in ginseng. Also, up-regulation of *SS*, *SE* and *DDS* coincided with endogenous JA biosynthesis in vanadate-treated ginseng (Huang and Zhong, 2013). Thus, JA has been regarded as the main signal transducer mediating *SS*, *SE* and *DDS* gene expression to enhance biosynthesis of ginsenosides in ginseng. However, the molecular link between the JA biosynthesis and ginsenoside production in ginseng plants is not clear.

JAs, including JA, JA methyl ester, JA amino acid conjugates and further JA metabolites, belong to the oxylipins, oxygenated compounds that are essential signaling molecules in growth and development and in responses to environmental changes. Oxylipin biosynthesis is initiated by the enzyme lipoxygenase (LOX; EC1.13.11.12), which is ubiquitous in plants and mammals and catalyses the hydroperoxidation of polyunsaturated fatty acids for further conversion into volatile aldehydes and JAs in plants (Mosblech *et al.*, 2009), into diols and lactones in fungi (Tsitsigiannis and Keller, 2007), and into lipoxins and leukotrienes in mammals (Samuelsson *et al.*, 1987; Sigal *et al.*, 1994). In plants, two types of LOX, 13-LOX and 9-LOX, catalyse the formation of two different isomer products, (13*S*)- and (9*S*)-hydroperoxyoctadecadienoic acids (13- and 9-HPOD) by introducing molecular oxygen at either carbon atom 13 or carbon atom 9, respectively, of the hydrocarbon backbone (Wasternack, 2007). The activity of 13-LOX is inhibited by diethyldithiocarbamic acid (DIECA) by oxidation of the products of 13-LOX, thereby reducing the substrate pool of 13-HPOD for JA production (Saltveit *et al.*, 2005) (see Supplementary Fig. S1A at *JXB* online).

Although JAs have been known as the elicitor signal for production of plant secondary metabolites, particularly the biosynthesis of ginsenosides (Zhao *et al.*, 2005; Rahimi *et al.*, 2014; Devi Balusamy *et al.*, 2015), this is the first report to show that controlling the JA pool (endogenous JA) using genetic engineering of a JA pathway gene subsequently increased triterpene ginsenoside levels. In this study, we show that *PgLOX6* encodes a lipoxygenase that is required for JA biosynthesis and promotes the expression of ginsenoside biosynthetic genes in *P. ginseng*. Overexpression of *PgLOX6* in the ginseng plant causes overproduction of JA, providing a new approach for increasing the production of ginsenosides.

## Materials and methods

### Plant materials and growth conditions

The Columbia ecotype of *Arabidopsis thaliana* was used as a model plant in this study. SALK_017873C was purchased from the Arabidopsis stock center (http://www.Arabidopsis.org/). Seeds were surface-sterilized and then sown on ½× Murashige and Skoog (MS) medium (Duchefa Biocheme, The Netherlands) containing 1% sucrose, 0.5 g l^–1^ 2-[*N*-morpholino]ethanesulphonic acid (MES), pH 5.7 with KOH, and 0.8% agar. Two-day cold-treated seeds were germinated under a long-day condition of 16h light–8h dark at 23 °C. Transformants were selected on hygromycin-containing plates (50 μg ml^–1^). Ten-day-old seedlings were transplanted into soil and allowed to grow for up to 5 weeks under the same light–dark conditions. For RNA extraction and metabolite analysis, leaves from 5-week-old plants were collected. Arabidopsis *pPgHMGR1::GUS*, *pPgHMGR2::GUS*, *pPgSE1::GUS*, *pPgSE2::GUS* and *pPgDDS::GUS* (Kim *et al.*, 2014) together with *pPgLOX6::GUS* lines were grown on ½MS solid medium for 1 week. The seedling leaves were pricked with a needle and harvested after 24h for β-glucuronidase (GUS) histochemical analysis.

### Ginseng materials and treatment

*P. ginseng* adventitious roots were collected from Ginseng Bank, Kyung Hee University and grown for 1 month in liquid MS medium (Murashige and Skoog, 1962) supplemented with 2mg l^–1^ indole-3-butyric acid (IBA) at 25 °C. The roots were maintained by regular subculture every 4 weeks. MJ was dissolved in a stock solution and microfiltered (0.2 µm). MJ (100 µM) was added to 4-week-old subcultured adventitious roots and sprayed onto leaves of 3-year-old ginseng plants. Cultures were harvested at 72 and 48h after treatment, respectively. The control plants were treated with ethanol. Different organs of 3-year-old healthy ginseng plants (flower, pedicel, peduncle, secondary leaf, primary leaf, petiole, stem, hypocotyl, rhizome and root) were collected from a ginseng field in Kyung Hee University, Korea. The plant material was immediately frozen in liquid nitrogen and stored at –70 ºC until required.

### Identification of genes and sequence analysis

A cDNA library was constructed (Sathiyamoorthy *et al.*, 2009) and a homologous sequence of LOX EST was searched in the GenBank databases using a BLASTX algorithm. We identified and selected the LOX gene based on the open reading frame (ORF) of the specific protein via the BlastX program (NCBI). ClustalX with default gap penalties was used to perform multiple alignments of proteins isolated from *P. ginseng* and previously registered in other species. A phylogenetic tree was constructed by the neighbor-joining method, and the reliability of each node was established by bootstrap methods using MEGA 6 software. Identification of conserved motifs within LOXs was accomplished with MEME (Bailey *et al.*, 2009) and MotifScan (http://myhits.isbsib.ch/cgi-bin/motif_scan). Chloroplast localization was inspected by ChloroP (Emanuelsson *et al.*, 1999) and proposed signal peptide was predicted from PSORT (http://ipsort.hgc.jp/index.html).

### RNA extraction and quantitative RT-PCR analysis

Total RNA was extracted from adventitious roots of *P. ginseng* using the RNeasy mini kit (Qiagen, Valencia, CA, USA). For RT-PCR, 1 μg of total RNA was used as a template for reverse transcription using oligo (dT)_15_ primer (0.1mM) and avian myeloblastosis virus (AMV) reverse transcriptase (10 U µl^–1^) (Intron Biotechnology, Inc., South Korea) according to the manufacturer’s instructions. Real-time quantitative PCR was performed using 100ng of cDNA in a 10 µl reaction volume using iQ^TM^SYBR^®^ Green Supermix. Gene-specific primers listed in Supplementary Table S1 were used to perform quantitative RT-PCR. The thermal cycler conditions recommended by the manufacturer were used as follows: 10min at 95 ºC, followed by 40 cycles of 95 ºC for 10s, 58 ºC for 10s, and 72 ºC for 20s. The fluorescent product was detected at the last step of each cycle. The relative quantity of the gene transcription levels was obtained using the Bio-Rad CFX connect Real-Time PCR Detection System, and calculated using the comparative cycle threshold (*C*_t_) method according to the manufacturer’s instructions for normalizing data. A constitutively expressed *β-actin* gene was used as internal reference. Three independent experiments were performed. The primer efficiencies were determined according to the method of Livak and Schmittgen (2001) to validate the ΔΔ*C*_t_ method. The observed slopes were close to zero, indicating that the efficiencies of the gene and the internal control *β-actin* were equal.

### Transgene constructs

To visualize the subcellular localization pattern of *PgLOX6*, cDNA sequences of *PgLOX6* were cloned into pCAMBIA1390 vector containing *cauliflower mosaic virus* (*CaMV*) *35S promoter and enhanced CFP* (*eCFP*). The *PgLOX6* cDNA was amplified using primers with *Sal*I and *Eco*RI sites (5′-AT *GTC GAC* ATG TTA AAC TCT CAG CTT CAC-3′ and 5′-CG *GAA TTC* AAT GGA AAT GCT ATT AGG-3′). The promoter region of genomic *PgLOX6* (1129 nucleotide) was amplified with *Pst*I and *Sal*I sites (5′-AT *CTG CAG* GAT GAA AAC CGT CG-3′ and 5′-GA *GTC GAC* GAG AGT TTA ACA TTG AGT TAC-3′) and cloned into pCambia 1300 vector containing the *GUS* gene.

All transgene constructs were confirmed by nucleotide sequencing before plant transformation. The constructs were transformed into Arabidopsis using *Agrobacterium tumefaciens* C58C1 (pMP90) (Bechtold and Pelletier, 1998). The insertion of transgenes in the transformants was confirmed by confocal microscopy or PCR analysis of the genomic DNA from the transformants. Homozygous plants with a 3:1 segregation ratio on the antibiotic plates were selected for further analyses. For each construct, 20–50 T1 independent lines were obtained, and chosen lines were analysed for the phenotypic significance of transgenes.

### Observation of reporter gene expression

The fluorescence from reporter protein was observed by confocal laser scanning microscopy (LSM 510 META, Carl Zeiss, Jena, Germany). Cyan fluorescent protein (CFP) was detected using 458/475–525nm excitation/emission filter sets, respectively. Fluorescence images were digitized with the Zeiss LSM image browser.

### Transformation into ginseng

Zygotic embryos of *P. ginseng* seeds after harvest (provided by Ginseng Bank) were stratified in humidified sand to mature for 3 months at 5 °C. After stratification, the seeds, in which zygotic embryos were in a mature state (4mm in length), were immersed in 70% ethanol for 1min, surface-sterilized in 2% NaOCl for 15min, and rinsed three times with sterilized distilled water. After carefully dissecting the zygotic embryos, they were placed on MS basal medium containing 3% sucrose and 0.8% agar. Five- to seven-day-grown zygotic embryos were excised transversely and cotyledon explants were used for transformation. They were dipped in the bacterial solution for 15min, subsequently blotted with sterile filter paper, and co-cultivated with *A. tumefaciens* C58C1 on MS medium containing 3% sucrose and 1 μg ml^–1^ 2,4-dichlorophenoxyacetic acid (2,4-D) for 2 d.

Thereafter, the explants were cultured on selection MS medium with 3% sucrose, 1 μg ml^–1^ 2,4-D, 0.5 μg ml^–1^ 6-benzylaminopurine (BA), 500 μg ml^–1^ cefotaxime and 50 μg ml^–1^ hygromycin. After subculture six times every 2 weeks on selection medium, the surviving cotyledons producing callus were cultured in the same medium without antibiotics and adventitious roots were induced from callus on B5 medium with 3% sucrose and 3mg l^–1^ IBA. Transgenic lines were generated and adventitious roots were excised from the maternal explants and subcultured in liquid B5 medium with 3% sucrose and 2mg l^–1^ IBA every 5 weeks.

### GUS histochemical analysis

Four-day-old seedlings and 50-d-old plants were treated with the indicated chemical for 3h before visualizing GUS activity. GUS staining was performed by incubating whole seedlings in the staining buffer containing 1mM 5-bromo-4-chloro-3-indoyl-β-D-glucuronic acid cyclohexylammonium salt (X-Gluc, Duchefa Biocheme, The Netherlands), 0.1 M NaH_2_PO_4_, 0.1% Triton X-100, and 0.5mM potassium ferri- and ferrocyanide at 37 °C until the blue color appeared (1–3h). Stained seedlings were cleared in 70% ethanol for 2h and 100% ethanol for 2h. At the final step of dehydration, samples were sequentially exposed to 10% (v/v) glycerol–50% (v/v) ethanol and 30% (v/v) glycerol–30% (v/v) ethanol. Seedlings were photographed under a microscope (ZEISS Axio Observer D1, Germany).

### LOX activity assay

Protein extracts were prepared from Arabidopsis leaves, which were homogenized with 1mL of 0.1 M sodium phosphate buffer (pH 6.5) using a pestle and mortar. The homogenate was centrifuged at 15 000*g* for 30min at 4ºC, and the resulting supernatant was used as the enzyme source. Protein concentrations of crude fractions were determined with a dye-binding protein assay kit (Bio-Rad) following the manufacturer’s instructions, with bovine serum albumin as a standard. LOX activity in the soluble fractions was spectrophotometrically measured by monitoring the increase of the conjugated diene hydroperoxide at *A*_234_ at 25 ºC for 10min (Richard *et al.*, 1992; Rancé *et al.*, 1998). The assay mixture included 0.1 M sodium phosphate buffer (pH 6.5), 0.1mM linoleic acid (Sigma-Aldrich), and 5–10mL of resuspended enzyme in a total volume of 1.2mL. The enzyme reaction was initiated by adding protein extracts from plants, and the change in absorbance was recorded. LOX activity is expressed as Δ*A*_234_ mg^–1^ protein. Linolenic acid was from Sigma-Aldrich.

### Ginsenoside extraction and analysis

Ginsenosides were extracted according to the method described by Kim *et al.* (2014). One gram aliquot of milled powder of freeze-dried roots was extracted twice for 1h by refluxing with 80% methanol at 70 ºC. The filtered extractions were evaporated using a rotary evaporator. The residue was dissolved in distilled water, followed by fractionation with water-saturated *n*-butanol. The butanol fractionation was performed two times. The butanol layers were combined and then evaporated to obtain the ginsenosides. Each sample was dissolved in methanol, filtered once through a 0.45 µm filter before high performance liquid chromatography (HPLC) analysis. The HPLC separation was performed on a C18 column (5 µm, 4.6×250mm) with water and acetonitrile as the mobile phase. The time and ratios of water and acetonitrile followed the protocol of Kim *et al.* (2014). The flow rate of the mobile phase was 1.6ml min^–1^, and ginsenosides were monitored at a wavelength of 203nm. Each ginsenoside was compared with an authentic ginsenoside sample purchased from ChromaDex Inc. (Irvine, CA, USA). Quantitative analysis was performed with a one-point curve method using external standards of authentic ginsenosides.

### Extraction and analysis of endogenous JA

Fresh samples (0.5g) were frozen in liquid nitrogen and ground to a fine powder using a mortar and pestle. Following the addition of 0.6ml of methanol, the homogenates were mixed and kept at 4 °C overnight, then centrifuged at 4800 *g* for 10min. The supernatant was transferred to a new 5ml glass tube, and the residue was re-extracted with 0.2ml of methanol; 3ml of double-distilled H_2_O was added to the combined extracts, and the solution was applied onto a Waters Sep-pak C18 cartridge. The cartridge was washed with 0.2ml of 20% methanol and 0.25ml of 30% methanol. The extract was eluted from the cartridge with 0.3ml of 100% methanol and collected for analysis. Liquid chromatography–tandem mass spectrometry (LC-MS/MS) measurements were performed as described by Liu *et al.* (2010). JA and MJ were purchased from Sigma-Aldrich.

### Extraction and quantification of triterpenes

Using the method from Kim *et al.* (2014), freeze-dried plant material (rosette leaves, 200mg) was powdered and then extracted twice with 1ml CHCl_3_–MeOH (7:3) at room temperature. 5-α-cholestane (20 μg) was used as the internal standard. The extract was dried in a rotary evaporator and saponifed with 1.5ml each of MeOH and 20% aqueous KOH for 1h at 80 °C to hydrolyse sterol esters. After saponification, 1.5ml each of MeOH and 4 M HCl was added for 1h at 80 °C and these reaction mixtures were extracted three times with 4ml of hexane. When the phases separated, sterols in the hexane layer were evaporated to dryness. The residue was trimethylsilylated with pyridine and *N*,*O*-bis(trimethylsilyl)trifluoroacetamide (BSTFA)+1% trimethylchlorosilane (TMCS) (1:1) at 37 °C for 90min and analysed by gas chromatography–mass spectrometry (GC-MS).

Capillary GC-MS analysis was performed using a mass spectrometer (HP 5973 MSD) connected to a gas chromatograph (6890A, Agilent Technologies) with a DB-5 (MS) capillary column (30 m×0.25mm, 0.25 μm film thickness). The analytical conditions were as follows: electron ionization (EI), 70eV; source temperature, 250 °C; injection temperature, 250 °C; column temperature program: 80 °C for 1min, then raised to 280 °C at a rate of 10 °C min^–1^, and held at this temperature for 17min; post-temperature, 300 °C; carrier gas, He; flow rate, 1ml min^–1^; run time, 38min; splitless injection. The endogenenous levels of squalene and β-amyrin were determined as the peak area ratios of molecular ions for the endogenous one and for the internal standard. Standards including squalene and triterpene (β-amyrin) were purchased from Sigma-Aldrich (USA).

## Results

### MJ triggers ginsenoside biosynthesis and gene expression

**Fig. 1.**
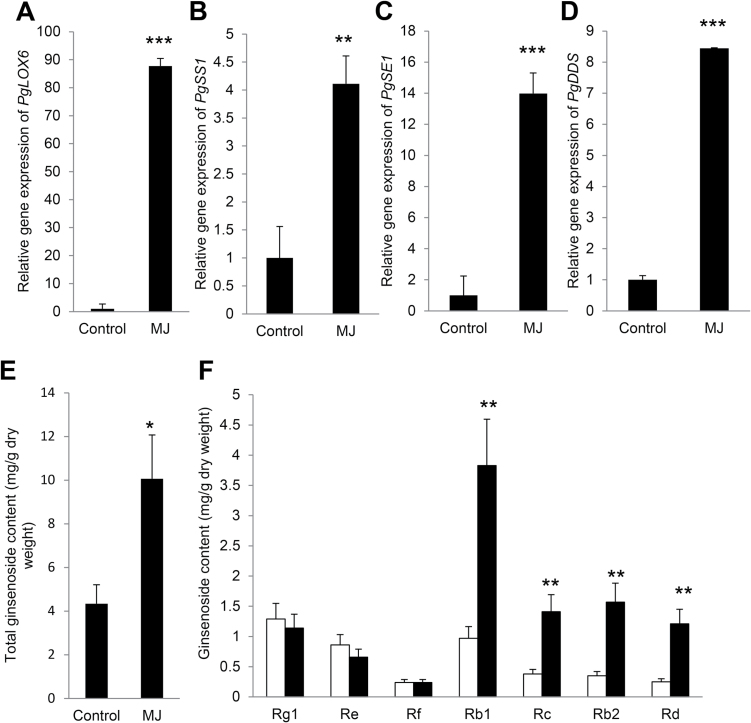
*PgLOX6* is associated with the production of ginsenoside. MJ (100 μM), an elicitor of ginsenoside production, was applied to 4-week-old adventitious roots for 3 d. (A–D) Induction of *PgLOX6* and ginsenoside-related genes by MJ treatment of ginseng adventitious roots. Steady-state transcription levels of *PgLOX6* and the ginsenoside biosynthetic genes *PgHMGR1*, *PgSS1*, *PgSE1*, and *PgDDS* were analysed using the same tissue. The *C*_t_ value for each gene was normalized to the *C*_t_ value for β-actin and calculated relative to a calibrator using the expression ${\text{2}}^{–\Delta \Delta C_{\text{t}}}.$ (E, F) Total contents of major ginsenosides (Rg1, Re, Rf, Rb1, Rb2, Rc, and Rd; milligrams per gram dry weight). Data represent the mean±SE of three independent replicates and were statistically analysed and compared with control using Student’s *t* test (*P<0.05; **P<0.01; ***P<0.001).

**Fig. 2.**
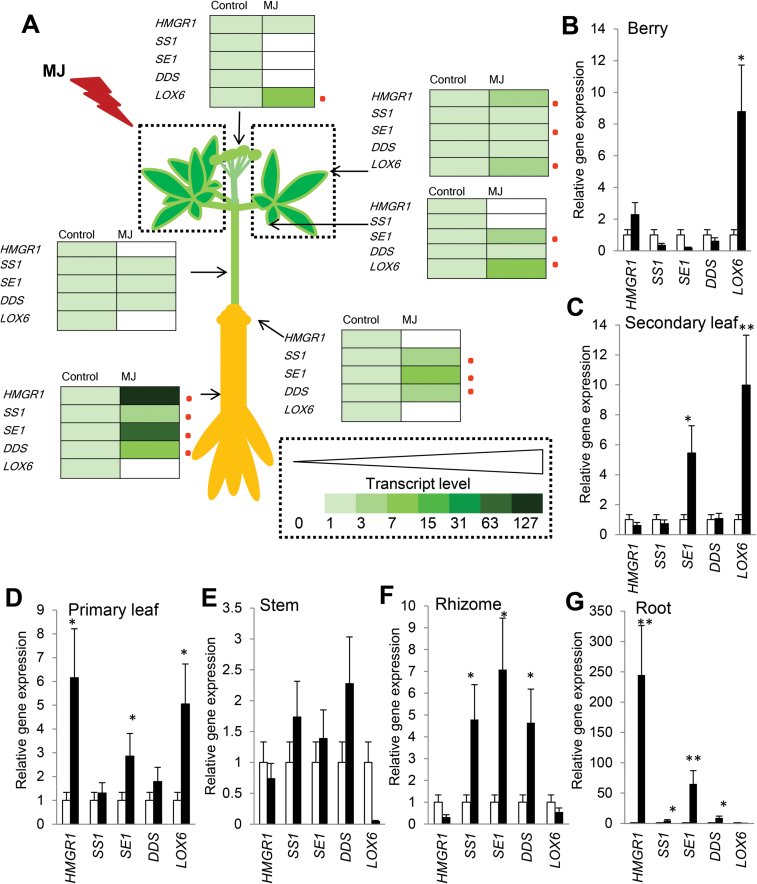
Induction of *PgLOX6* in aerial organs is associated with the expression of ginsenoside genes in underground organs. (A) MJ (100 μM), an elicitor of ginsenoside production, was applied to the leaf of 3-year-old ginseng for 2 d. (B–G) Steady-state transcription levels of *PgLOX6* and the ginsenoside biosynthetic genes *PgHMGR1*, *PgSS1*, *PgSE1*, and *PgDDS* were analysed using the same organs. The *C*_t_ value for each gene was normalized to the *C*_t_ value for β-actin and calculated relative to a calibrator using the expression ${\text{2}}^{–\Delta \Delta C_{\text{t}}}.$ Data represent the mean±SE of three independent replicates and were statistically analysed and compared with controls using Student’s *t* test (*P<0.05; **P<0.01). The circles show significant differences. (This figure is available in color at *JXB* online.)

To further reveal how JA promotes ginsenoside synthesis, firstly we applied a JA biosynthetic inhibitor, DIECA, to MJ-treated ginseng adventitious roots. The ginsenoside content of DIECA-treated roots decreased to 1.1mg g^–1^ dry weight (DW) compared with the control sample of 2.5mg g^–1^ DW and MJ-treated roots of 5.3mg g^–1^ DW (see Supplementary Fig. S1B). Furthermore, qRT-PCR analysis showed that the treatment by DIECA significantly decreased the expression of JA biosynthetic genes 72h after treatment, including allene oxide synthase (*PgAOS*), with activity in converting 13-LOX products to allene oxides, allene oxide cyclase (*PgAOC1*), which metabolizes allene oxides to (9*S*,13*S*)-12-oxo phytodienoic acid (OPDA), as well as ginsenoside synthetic genes such as *SS1*, *SE1*, and *DDS* (Supplementary Fig. S1C, D). By contrast, MJ-treated roots displayed 4.1-, 14-, and 8.4-fold increases of *PgSS1*, *PgSE1*, and *PgDDS*, respectively, compared with control, which coincided with higher expression of the JA pathway genes *PgLOX6* and *PgAOC1* (data not shown), 72h after MJ treatment (Fig. 1A–D). This led to a 2.3-fold increase of total ginsenoside level in 4-week-old ginseng adventitious roots, with a particularly obvious increase of PPD-type ginsenosides such as Rb1, Rb2, Rc, and Rd (Fig. 1E, F). These results suggest that JA is able to promote the transcription of ginsenoside-synthesizing genes, leading to an increase of JA biosynthesis in ginseng plants. To determine the expression profile of *PgLOX6* along with ginsenoside biosynthetic genes in response to MJ in whole organs of ginseng, we conducted qRT-PCR using 3-year-old ginseng after 2 d of MJ application to the leaves (Fig. 2A). In berries *PgLOX6* was upregulated compared with control, whereas in leaf *PgHMGR1* and *PgSE1* were upregulated along with *PgLOX6* (Fig. 2B–D). In the stem *PgDDS* mRNA was more abundant compared with control (Fig. 2E). Interestingly, significant expression of *PgSS1*, *PgSE1*, and *PgDDS* was identified in the treated rhizome and roots (Fig. 2F, G). Therefore, LOX expression was dominant in the aerial parts of the plant, which differs from the strong expression of ginsenoside biosynthetic genes in underground tissues. These results can be correlated with a previous report that proved the hypothesis that JA functions in the transfer of the damage signal from the shoot to the root, which stimulates root nicotine synthesis (Baldwin *et al.*, 1994). However, further experiments are required to confirm this hypothesis for the localization of ginsenoside biosynthesis in ginseng.

### Sequence characterization of *PgLOX6*

Each plant species has multiple LOX members with various biological functions (Andreou and Feussner, 2009). From the EST library constructed using ginseng adventitious root treated with MJ, we observed six putative *LOX* sequences, named *PgLOX1*, *PgLOX2*, *PgLOX3*, *PgLOX4*, *PgLOX5*, and *PgLOX6* (Sathiyamoorthy *et al.*, 2009; Bae *et al.*, 2016; this study). To understand the phylogenetic position of these six *PgLOX* genes and gain information on their potential function, we performed BLAST using the full-length sequences of these six PgLOXs as the query sequences. A total of 34 closest annotated sequences were obtained from 19 different organisms including Arabidopsis, fungi, animals, and prokaryotic organisms, but not from archaea (Li *et al.*, 2010). Clustal X and the MEGA 6 were used for constructing a phylogenetic tree based on LOX amino acid sequences (see Supplementary Figs S2 and S3A). MEME analysis found three conserved motifs in plant LOXs located in the LOX catalytic domain; homologs in animals contained Motif 1 and Motif 2, and prokaryotes and fungi had only Motif 1 (Supplementary Figs S2 and S4). Generally, the distribution of conserved motifs among the plant LOXs was very similar, but quite different from that of the fungi, animals and prokaryote, as expected from the evolutionary distances for LOXs (Li *et al.*, 2006). From the phylogenic tree, we classified the plant LOXs into two subfamilies of 13-LOX and 9-LOX. At the bottom of the substrate binding pocket, a space-filling histidine or phenylalanine residue occurs in nearly all plant 13-LOXs, which can determine enzyme positional specificity; in contrast, all plant 9-LOXs have a small valine residue at this amino acid position (Andreou and Feussner, 2009). Along with 13-LOXs (AtLOX2, AtLOX3 AtLOX4, and AtLOX6) encoded by the genome of Arabidopsis and other plant species that function in JA biosynthesis (Royo *et al.*, 1996; Cheng *et al.*, 2006; Chauvin *et al.*, 2013; Yan *et al.*, 2013), *PgLOX4*, *PgLOX5*, and *PgLOX6* were classified in the subfamily of 13-LOXs, whereas *PgLOX1*, *PgLOX2*, and *PgLOX3* together with 9-LOXs from Arabidopsis (AtLOX1 and AtLOX5) and other plant species belonged to the subfamily of 9-LOXs.

As shown in Supplementary Fig. S5), among three 13-LOXs in ginseng, a higher transcription level of PgLOX6 was found at 6 and 12h after MJ treatment (50 μM) of 1-month-old ginseng seedling compared with PgLOX4 and PgLOX5, which may suggest that PgLOX6 is early responsive to MJ treatment. Therefore, PgLOX6 was selected for further analysis; however, further experiments are required to investigate the functional role of other 13-LOXs (PgLOX4 and PgLOX5) in ginseng to support these results.

The coding sequence of *PgLOX6* is 2718bp in length and encodes a 906-amino-acid protein. The genomic DNA sequence of *PgLOX6* retrieved from the ginseng genome database (http://im-crop.snu.ac.kr/new/index.php) is 7965bp in length, containing nine exons and eight introns (see Supplementary Figs S3B and S6). Sequence alignment of PgLOX6 with other plant homologs revealed that it shared two conserved domains (Supplementary Fig. S7) that are characteristic of the LOX family (Andreou and Feussner, 2009). The catalytic domain of LOX contains the iron binding region including three histidines, one asparagine and the carboxy group of the C-terminal isoleucine, which is consistent with other plant LOXs (Andreou and Feussner, 2009). The N-terminal sequence (30 aa) of PgLOX6 was predicted to be a plastidial transit peptide by iPSORT and ChloroP (Supplementary Fig. S7), suggesting a chloroplast localization of PgLOX6, which is in agreement with the role of chloroplasts in synthesizing JA (Hause *et al.*, 2003). However, there was no homology among the N-termini of the LOXs as these regions are transit peptides whose amino acid sequences show much variation. From the alignment, it was revealed that the deduced amino acids of PgLOX6 shared a higher sequence homology with 13-LOX proteins in plants.

As shown in Supplementary Fig. S8, LOX activity was analysed in extract of isopropyl β-D-1-thiogalactopyranoside (IPTG)-induced transgenic bacteria with optimal function at pH 6 and higher LOX activity with 0.2mM of linoleic acid as the substrate.

### Expression pattern of *PgLOX6*

**Fig. 3.**
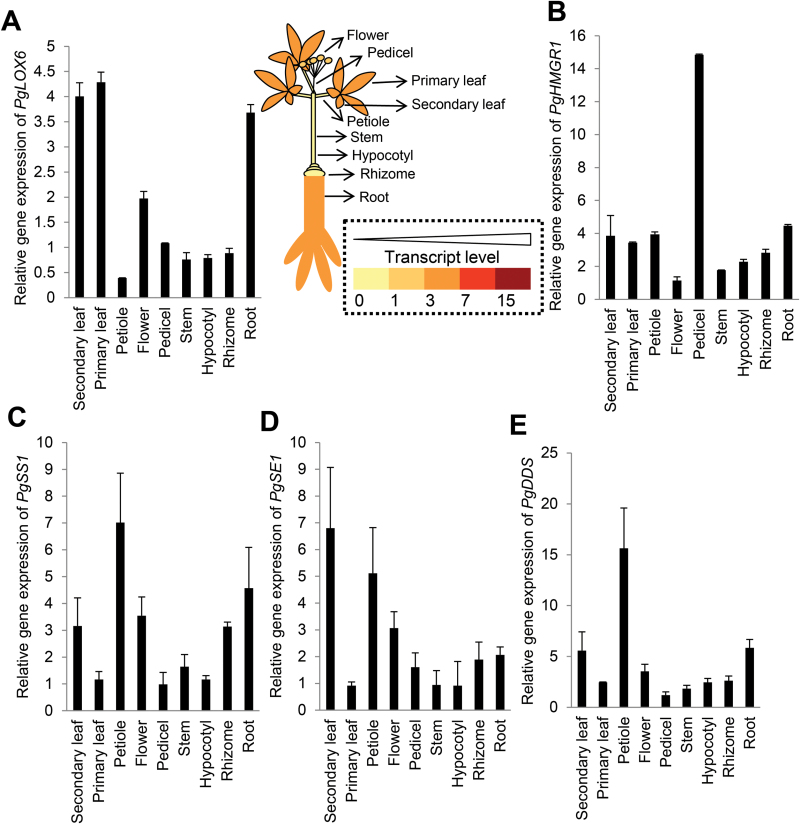
Organ-specific expression pattern of *PgLOX6* along with ginsenoside biosynthetic genes. Material from 3-year-old ginseng was used for qRT-PCR. (A–E) Steady-state transcription levels of *PgLOX6* and the ginsenoside biosynthetic genes *PgHMGR1*, *PgSS1*, *PgSE1*, and *PgDDS* were analysed using the same tissue. The *C*_t_ value for each gene was normalized to the *C*_t_ value for β-actin and calculated using the expression ${\text{2}}^{–\Delta \Delta C_{\text{t}}}.$ Data represent the mean±SE of three independent replicates. (This figure is available in color at *JXB* online.)

**Fig. 4.**
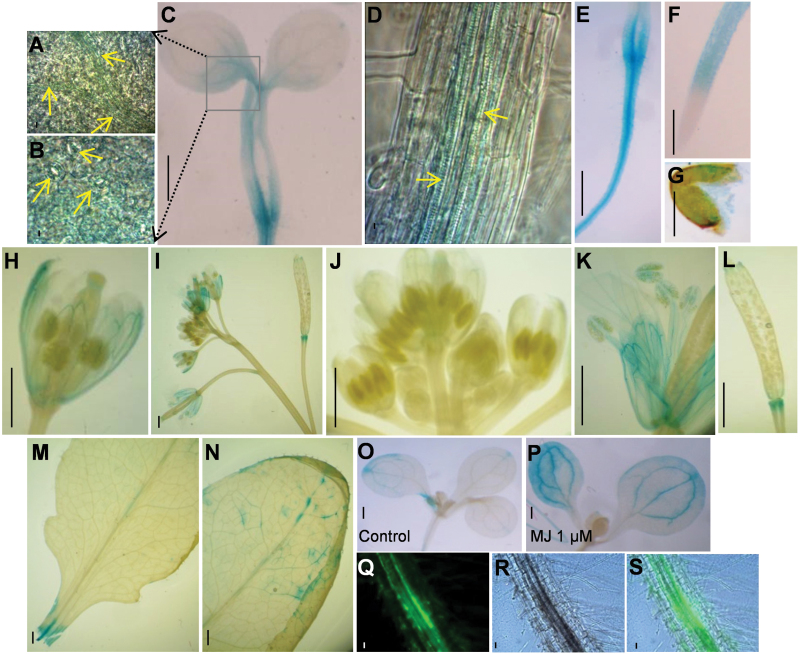
Vascular bundle expression of PgLOX6. Expression pattern of PgLOX6 by histochemical analysis of GUS expression in transgenic Arabidopsis plants harboring *pPgLOX::GUS* at different developmental stages. (A–G) GUS expression in 4-d-old germinated seedlings. (H–N) GUS expression in 50-d-old plant. (O, P) GUS expression in 4-d-old germinated seedlings treated with 1 μM MJ compared with control. (Q–S) localization of PgLOX6 tagged with cyan fluorescent protein. Fluorescence images were visualized by a confocal laser scanning microscope. Scale bars: 100 µm. (This figure is available in color at *JXB* online.)

To understand the function of *PgLOX6*, we performed expression pattern analysis of *PgLOX6* along with ginsenoside biosynthetic genes using 3-year-old ginseng plants (Fig. 3A–E). qRT-PCR analysis showed that *PgLOX6* was most highly expressed in leaf, root and flower (Fig. 3A), suggesting it was ubiquitously expressed in ginseng plants. Furthermore, we fused the promoter sequence of *PgLOX6* (–1129 to 1bp) to *GUS* to make a *pPgLOX6::GU*S construct (Fig. 4). Arabidopsis transgenic plants containing *pPgLOX6::GUS* showed the expression signal of GUS in most tissues of 4-d-old seedlings (Fig. 4A–G); it was particularly highly detected in the root vasculature, but restricted in the root tips (Fig. 4D–F). Furthermore, we observed *GUS* expression in leaf, especially in guard cells (Fig. 4B, C, M, N), consistent with the role of JA in modulating stomatal closure in response to pathogens (Montillet *et al.*, 2013). Furthermore, we observed the GUS signal in the filament, flower sepal (Fig. 4H–K) and junction between silique and pedicel of a 50-d-old plant (Fig. 4L), which corresponds with JA’s role in regulating flower development, pollen maturation and anther dehiscence in Arabidopsis and rice (Browse, 2009; Cai *et al.*, 2014). Besides the promoter activity analysis, we also observed the localization of PgLOX6 fused with cyan fluorescent protein (CFP) within the vascular bundles of roots in transgenic Arabidopsis (named *PgLOX6ox*; Fig. 4Q–S). In support of this, the JA biosynthetic enzymes such as LOX, AOS, and AOC were shown to be located within sieve elements connected to the companion cells of the phloem, allowing the long-distance transport of JA in plants (Hause *et al.*, 2003).

To determine the correlation of LOX and ginsenoside biosynthesis related genes, the *pPgLOX6::GUS*, *pPgHMGR1::GUS*, *pPgHMGR2::GUS*, *pPgSE1::GUS*, *pPgSE2::GUS* and *pPgDDS::GUS* lines were wounded (see Supplementary Fig. S9). Similar to *pPgLOX6::GUS* plants, the transformed Arabidopsis with ginsenoside-related gene promoter fused with *GUS* showed a higher *GUS* expression pattern at the wounded sites, suggesting that *PgLOX6* and ginsenoside-related genes are responsive to wounding.

However, contrary to these results, when the SALK line (SALK_017873C) of *AtLOX4* (ortholog sequence of *PgLOX6*; see Supplementary Fig. S10A, B), a *lox4* mutant with decreased LOX activity and MJ content (Supplementary Fig. S10C, D), was wounded, the squalene content was decreased compared with WT (Supplementary Fig. S10E). At this time, the expression of *AtSE* and *Atβ-AS* genes did not show any difference compared with WT (Supplementary Fig. S10F, G). The interesting fact is the reduced mRNA level of *AtMYC2* compared with untreated *lox4* mutant (Supplementary Fig. S10H). Thus, we can suggest that the JA pathway can regulate terpenoid biosynthetic genes through transcription regulators, which is matched in the higher expression of transcription factors in *PgLOX6*-overexpressing ginseng along with increased ginsenoside level. This result leads to the hypothesis that the loss of PgLOX6 function may cause decreased level of squalene precursor for ginsenoside biosynthesis, which would need to be proved by silencing PgLOX6 in *P. ginseng*.

### Overexpression of *PgLOX6* in Arabidopsis promotes the production of JA and triterpene precursor

**Fig. 5.**
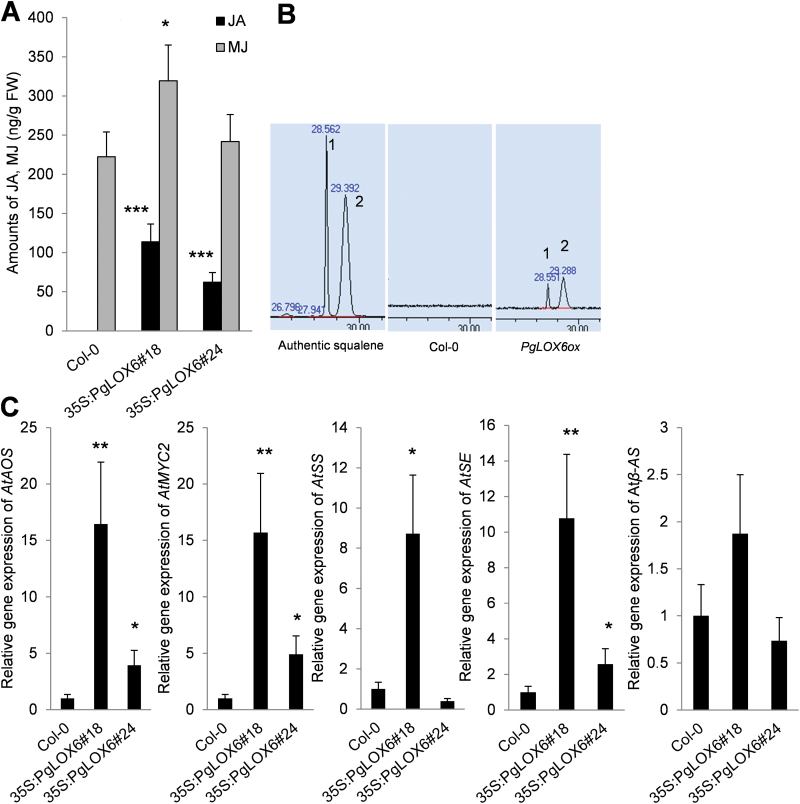
PgLOX6-derived JA results in squalene production. Arabidopsis transgenic plants expressing *PgLOX6* and Col-0 were grown and used for analysis. (A) Quantification of endogenous JA and MJ level in Arabidopsis using LC-MS. (B) Quantification of endogenous squalene in Arabidopsis using GC-MS (A, authentic squalene; B, Col-0; C, *PgLOX6ox*). (C) Steady-state transcription level of *AtAOS*, *AtMYC2*, *AtSS*, *AtSE*, and *Atβ-AS* in transgenic Arabidopsis expressing *PgLOX6*. The *C*_t_ value for each gene was normalized to the *C*_t_ value for β-actin and calculated relative to a calibrator using the expression ${\text{2}}^{–\Delta \Delta C_{\text{t}}}.$ Data represent the mean±SE of three independent replicates and were statistically analysed and compared with controls using Student’s *t* test (*P<0.05; **P<0.01; ***P<0.001).

To reveal whether LOX is responsible for JA biosynthesis, we measured the JA and MJ contents in Arabidopsis transgenic lines overexpressing *PgLOX6* (*PgLOX6ox*). As expected, transgenic lines had a higher level of JA compared with control (Fig. 5A), suggesting the involvement of *PgLOX6* in JA biosynthesis.

To determine whether PgLOX6 contributes to the metabolic changes in *PgLOX6ox* lines, we examined the expression of triterpene-related genes and the amounts of triterpenes extracted from the rosette leaves of 4-week-old plants. GC-MS analysis revealed that the content of squalene, a precursor of triterpenes, was increased in *PgLOX6ox* transgenic Arabidopsis leaves (Fig. 5B). Moreover, qRT-PCR results showed a significant increase in endogenous transcripts of triterpene-related genes including *AtSS* and *AtSE* in *PgLOX6ox* lines compared with WT (Fig. 5C).

### Overexpression of *PgLOX6* in ginseng increases the production of JA and ginsenosides

**Fig. 6.**
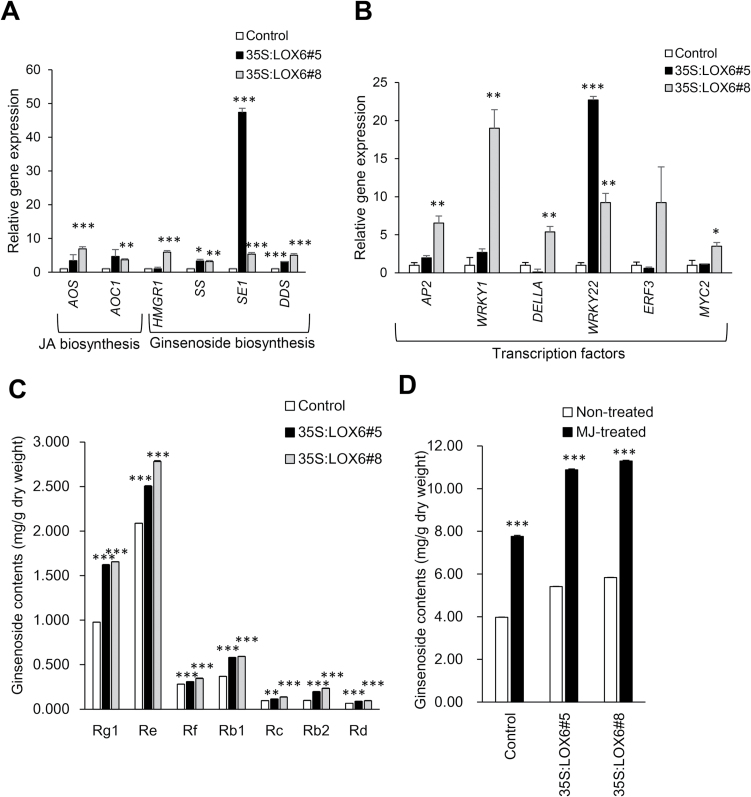
Overexpression of *PgLOX6* results in higher production of ginsenoside. Transgenic adventitious roots of ginseng overexpressing *PgLOX6* were used for analysis. (A, B) Steady-state transcription level of JA biosynthetic genes *PgAOS* and *PgAOC1*, ginsenoside biosynthetic genes *PgHMGR1*, *PgSS1*, *PgSE*, and *PgDDS*, and transcription factors *PgAP2*, *PgWRKY1*, *PgDELLA*, *PgERF3*, and *PgMYC2* in transgenic ginseng roots expressing *PgLOX6*. The *C*_t_ value for each gene was normalized to the *C*_t_ value for β-actin and calculated relative to a calibrator using the expression ${\text{2}}^{–\Delta \Delta C_{\text{t}}}.$ (C) Quantitative analysis of major ginsenoside (Rg1, Re, Rf, Rb1, Rb2, Rc, and Rd) contents in control and transgenic ginseng adventitious roots were analysed by HPLC. (D) Quantitative analysis by HPLC of total ginsenoside contents in control and transgenic ginseng adventitious roots treated with 100 μM MJ 24h after treatment. Data represent the mean±SE of three independent replicates and were statistically analysed and compared with controls using Student’s *t* test (*P<0.05; **P<0.01; ***P<0.001).

To evaluate the role of *PgLOX6* in synthesizing ginsenosides in ginseng plants, *PgLOX6* was constitutively overexpressed under the CaMV35S promoter in ginseng adventitious roots. Transgenic *PgLOX6ox* adventitious roots showed increased expression of JA synthetic genes such as *PgAOS* and *PgAOC1*; however, there was no significant increase of JA and MJ in transgenic roots compared with the control. The transgenic *PgLOX6ox* ginseng plants significantly activated JA production 24h after treatment with 100 μM MJ compared with the control (see Supplementary Table S2). Moreover, the expression of ginseng synthetic genes *PgHMHGR1*, *PgSS1*, *PgSE1*, and *PgDDS* was higher in *PgLOX6ox* ginseng adventitious root compared with control (Fig. 6A). The total ginsenoside content was 5.41 and 5.83mg g^–1^ DW in *PgLOX6ox* ginseng no. 5 and no. 8, respectively, which is higher than control roots (3.97mg g^–1^ DW); in particular, ginsenosides Rb2, Rg1, and Rb1 were 2.4, 1.7, and 1.6 times greater than the amount in the control (Fig. 6C). Furthermore, when we treated *PgLOX6ox* with MJ, total ginsenoside level was increased 1.4 times compared with MJ-treated non-transgenic control ginseng, and was 2.8 times higher than non-treated, non-transgenic control (Fig. 6D). This result can be correlated with the function of LOX in enhancement of JA’s role in the ginsenoside biosynthesis pathway.

## Discussion

Ginseng as a folk and conventional medicine has been used for the treatment of different diseases for over 5000 years. The fundamental pharmacologically active ingredient of ginseng, ginsenoside, has wide beneficial activity (Radad *et al.*, 2006). Activation of ginsenoside biosynthetic genes and ginsenoside accumulation are mediated by elicitor-induced signaling molecules (Rahimi *et al.*, 2015*a*). JA is the well-known signaling molecule that has been shown to be mainly involved in ginsenoside accumulation. Understanding the mechanism of JA synthesis and how to increase ginsenoside accumulation will be helpful for commercial production of ginsenosides. Here we showed that PgLOX6, a 13-LOX enzyme, is responsible for JA biosynthesis. Transgenic plants overexpressing *PgLOX6* in Arabidopsis increased the amount of JA and MJ, as well as the expression of triterpene biosynthetic genes and the precursor of ginsenosides, squalene. In particular, transgenic ginseng roots overexpressing *PgLOX6* showed increased levels of ginsenoside. These results showed the role of PgLOX6 in biosynthesizing JA and promotion of ginsenoside production, providing a molecular tool for increasing ginsenoside production.

### Ginsenosides are actively biosynthesized in vascular bundles

Ginsenoside biosynthesis is catalysed through the function of three different essential enzymes, SS, SE, and DDS (Kim *et al.*, 2015). We have shown the higher expression of *PgSS1*, *PgSE1* and *PgDDS* in petioles (Fig. 3C–E). By *in situ* hybridization analysis, *PgSS1*, *PgSS3*, *PgSE1*, and *PgSE2* were shown to be preferentially expressed in vascular bundle tissues and resin ducts in petioles (Kim *et al.*, 2015). Similarly, the expression of *pPgLOX6::GUS* and PgLOX6-CFP was detected at the vascular bundles, and MJ treatment of *pPgLOX6::GUS* showed higher GUS activity in veins of Arabidopsis (Fig. 4O, P). As was already demonstrated by immunocytological analysis, the enzymes of JA biosynthesis, including LOX, AOS, and AOC, are detected in sieve elements of tomato plants (Hause *et al.*, 2003). Expression of *PgLOX6* in vascular bundles can be associated with the vascular bundle-specific generation of JAs and supports the role of this gene in systemic wound signaling.

It was already known that the long-distance transport of assimilates is conducted from source leaves to non-photosynthesizing sink organs, such as roots, flowers and developing seeds, through the phloem (Lucas *et al.*, 2014). It was also already known that root epidermis contains a high level of ginsenosides, and that ginsenosides are mainly distributed in the oil canals of periderm and outer cortical regions but not in the xylem or pith in root tissues, which is different from the ginsenoside gene expression profile. Therefore, phloem and resin ducts were suggested to be the production sites of sterol and ginsenoside, which may accordingly move to other tissues, such as the epidermis (Kim *et al.*, 2015). Interestingly, the results of MJ treatment of the ginseng leaf were in contrast with the previous idea, showing a higher transcription level of ginsenoside genes in the root tissue. However, the *PgLOX6* mRNA level was significantly high in berry and leaf tissues, but weakly detected in stem, rhizome, and root. This result suggests the idea that the higher expression of *PgLOX6* in aerial tissues like berry and leaf could contribute to vascular bundle generation of JA, which may lead to the higher expression of ginsenoside genes such as *HMGR1*, *SS1*, *SE1*, and *DDS* in underground tissues like rhizome and root.

As was shown in Supplementary Fig. S4, chloroplast localization and plastidial signal peptide were shown for PgLOX6 and its homologs. There are several studies showing that organelles such as plastids, peroxisome, and vacuoles are involved in ginsenoside biosynthesis in leaves, which may occur along with JA biosynthesis through successive reactions in chloroplast and peroxisome (Poustka *et al.*, 2007; Yokota *et al.*, 2011).

### PgLOX6 contributes to ginsenoside overproduction

JAs are signaling molecules whose production is catalysed by several chloroplastic enzymes, 13-LOX, 13-AOS, and AOC, and finally by the peroxisomal oxophytodienoate reductase (OPR). There are several studies showing that the manipulation of endogenous JA level can alter secondary metabolism, which can be achieved through an elicitation strategy (Rahimi *et al.*, 2015*a*) or by genetic engineering of JA pathway genes. A previous study (Lu *et al.*, 2014) demonstrated that increased endogenous JA could significantly promote the biosynthesis of the sesquiterpenoid artemisinin in *AaAOC*-overexpression transgenic *Artemisia annua*. However, there are several reports of enhanced production of tanshinone and camptothecin in *AOC*-overexpression transgenic *Salvia miltiorrhiza* and *Camptotheca acuminate*, respectively (Gu *et al.*, 2012; Pi *et al.*, 2012). Also, enhanced production of PPD was observed by overexpression of Arabidopsis JA carboxyl methyltransferase (the MJ biosynthesis gene) in transgenic *P. ginseng* roots (Kim *et al.*, 2012).

LOX is the key enzyme in the JA biosynthetic pathway; however, the effect of this gene on secondary metabolism remains unknown. LOX is encoded by multiple LOX genes. In this study, a new 13-LOX, *PgLOX6*, was identified. To determine whether overexpressing the *LOX* gene could promote ginseng production through the engineered internal JA pool, *PgLOX6* was incorporated into Arabidopsis as a model plant and ginseng. Importantly, overexpressing *PgLOX6* in Arabidopsis and ginseng adventitious roots actually resulted in increased triterpenoid biosynthetic gene expression as well as squalene precursor and ginsenoside production, respectively. As predicted, PPD group ginsenoside accumulated at a higher level than that of the PPT group. Interestingly, MJ treatment of transgenic ginseng significantly enhanced JA and MJ, associated with 2.8-fold higher ginsenoside accumulation compared with control non-treated, non-transgenic plants, which is 1.4 times higher than the MJ-only treatment strategy on non-transgenic plants. The results of qRT-PCR showed that, compared with the control level, the transcript level of artemisinin biosynthetic pathway genes like *FPS*, *CYP71AV1*, and *DBR2* was increased significantly in *AaAOC*-overexpression transgenic *A. annua* (Lu *et al.*, 2014)*. SmAOC* overexpression significantly enhanced expression level of key genes involved in the biosynthetic pathway of diterpenes and phenolic acids (Gu *et al.*, 2012). Taken together, we conclude that the genetic manipulation of the JA pathway would be helpful for improving the production of valuable secondary metabolites; however, it is not clear how JA signaling promotes the secondary metabolism.

JA-mediated production of secondary metabolites was regulated by basic helix–loop–helix (bHLH) proteins in *Catharanthus roseus*, *Arabidopsis thaliana*, *Nicotiana tabacum*, and *N. benthamiana* (Lenka *et al.*, 2012). *MYC2* overexpression in Arabidopsis caused enhanced sesquiterpene emission and higher expression of sesquiterpene synthase genes (Hong *et al.*, 2012). Adventitious roots treated by MJ up-regulated transcription factor families like WRKY, MYB, AP2, NAP, G/HBF-1 and ERF, which coincided with ginsenoside accumulation (Subramaniyam *et al.*, 2014). Heterologous *PqWRKY1* overexpression in Arabidopsis regulated triterpene biosynthesis-related genes (Sun *et al.*, 2013). In this study, the potential candidates of *PgWRKY22*, *PgWRKY1*, *PgAP2*, *PgDELLA*, *PgERF3*, and *PgMYC2* were suggested as transcription factors regulating ginsenosides biosynthesis (Fig. 6B).

**Fig. 7.**
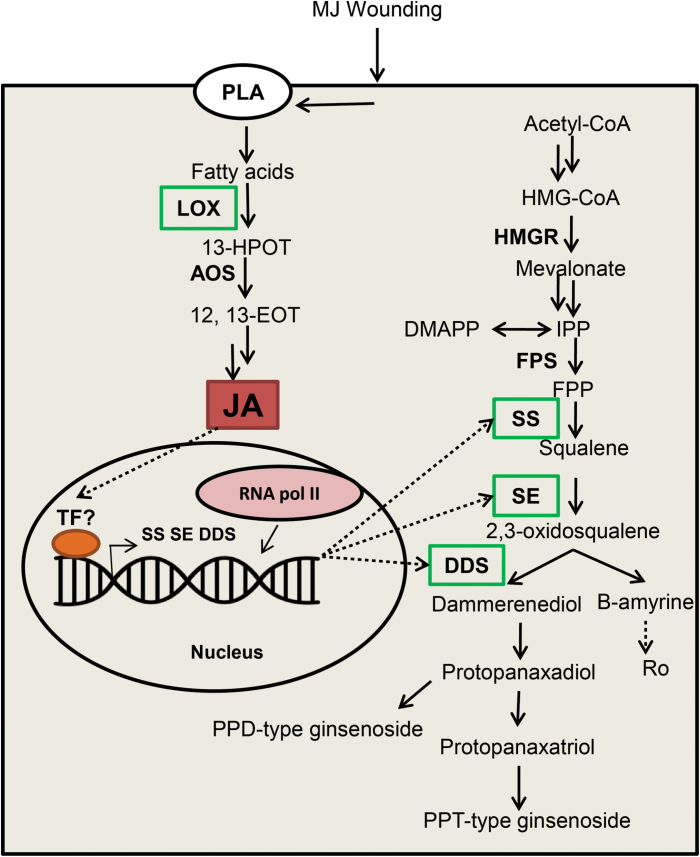
Proposed model for the regulation of ginsenoside biosynthesis by JA signaling pathways. LOX-derived JA can regulate triterpenoid saponin accumulation through the candidate transcription factors. (This figure is available in color at *JXB* online.)

Here, we propose a model to elucidate the mechanism of regulation of ginsenoside biosynthesis by LOX-derived JA (Fig. 7). Further investigation into the transcription regulatory mechanism of ginsenoside biosynthetic genes will provide valuable data for understanding JA-pathway-mediated secondary metabolism in ginseng and possibly help in manipulating ginsenoside accumulation.

## Supplementary data

Supplementary data are available at *JXB* online.

Figure S1. Suppression of ginsenoside biosynthesis by JA inhibition.

Figure S2. Structural features of PgLOX6.

Figure S3. Structural features of PgLOX6 compared with AtLOXs.

Figure S4. Conserved motifs among LOXs.

Figure S5. Induction of *PgLOX6* and other 13-LOXs by MJ treatment in 1-month-old ginseng seedling.

Figure S6. Genomic DNA sequence of *PgLOX6* with its promoter (–1317 to –1), and a deduced amino acid sequence.

Figure S7. Multiple alignment of the deduced amino acid sequences of PgLOX6 with homologous LOXs from other plants.

Figure S8. Biochemical assay of PgLOX6.

Figure S9. Effects of wounding on *pPgLOX6::GUS*, *pPgHMGR1::GUS*, *pPgHMGR2::GUS*, *pPgSE1::GUS*, *pPgSE2::GUS*, and *pPgDDS::GUS*.

Figure S10. Lack of LOX-derived JA decreased triterpene content in wounded Arabidopsis.

Table S1. Primers used in this study.

Table S2. *PgLOX6* contributes to JA production in MJ-treated transgenic roots.

Supplementary Data
