# *PgLOX6* encoding a lipoxygenase contributes to jasmonic acid biosynthesis and ginsenoside production in *Panax ginseng*

**DOI:** 10.1093/jxb/erw413

**Published:** 2016-10-31

**Authors:** Shadi Rahimi, Yu-Jin Kim, Johan Sukweenadhi, Dabing Zhang, Deok-Chun Yang

**Affiliations:** 1 Graduate School of Biotechnology and Ginseng Bank, College of Life Sciences, Kyung Hee University, Yongin, Republic of Korea; 2 Department of Crop Science, Chungbuk National University, Cheongju, Korea; 3 Department of Oriental Medicinal Biotechnology, College of Life Sciences, Kyung Hee University, Yongin, Republic of Korea; 4 State Key Laboratory of Hybrid Rice, Shanghai Jiao Tong University–University of Adelaide Joint Centre for Agriculture and Health, School of Life Sciences and Biotechnology, Shanghai Jiao Tong University, Shanghai, China; 5 School of Agriculture, Food and Wine, University of Adelaide, Waite Campus, Urrbrae, South Australia, Australia

*Journal of Experimental Botany* doi:10.1093/jxb/erw358

The authors of the above article would like to make two minor corrections:

1) The affiliations of the author Deok-Chun Yang were incorrectly given as institutions 2 and 3. This has been corrected to 1 and 3 in the article and above.
2) The iPET research support number given in the acknowledgements was incorrect and should read: iPET (112142-05-4-SB010). This has now been corrected in the article.

