# Supplementary material for: PgLOX6 encoding a lipoxygenase contributes to jasmonic acid biosynthesis and ginsenoside production in Panax ginseng
Source: J Exp Bot. 2016 Oct 6;67(21):6007–19. doi: 10.1093/jxb/erw358 (PMC5100016; doi:10.1093/jxb/erw358)
Supplement: Supplementary Data [file supp_67_21_6007__index.html]

 PgLOX6 encoding a lipoxygenase contributes to jasmonic acid biosynthesis and ginsenoside production in Panax ginseng — PgLOX6 encoding a lipoxygenase contributes to jasmonic acid biosynthesis and ginsenoside production in Panax ginseng — PgLOX6 encoding a lipoxygenase contributes to jasmonic acid biosynthesis and ginsenoside production in Panax ginseng — Supplementary Data 

# *PgLOX6* encoding a lipoxygenase contributes to jasmonic acid biosynthesis and ginsenoside production in *Panax ginseng*

## Supplementary Data

Data files

- supplementary\_figures\_S1\_S10\_Tables\_S1\_S2.pdf - Supplementary Data
