# Supplementary material for: PgLOX6 encoding a lipoxygenase contributes to jasmonic acid biosynthesis and ginsenoside production in Panax ginseng
Source: J Exp Bot. 2016 Oct 6;67(21):6007–19. doi: 10.1093/jxb/erw358 (PMC5100016; doi:10.1093/jxb/erw358)
Supplement: Supplementary Data [file supp_erw358_supplementary_figures_S1_S10_Tables_S1_S2.pdf]

***PgLOX6* encoding a lipoxygenase contributes to jasmonic acid biosynthesis and ginsenoside production in *Panax ginseng***

Shadi Rahimi, Yu-Jin Kim, Johan Sukweenadhi, Dabing Zhang, Deok-Chun Yang

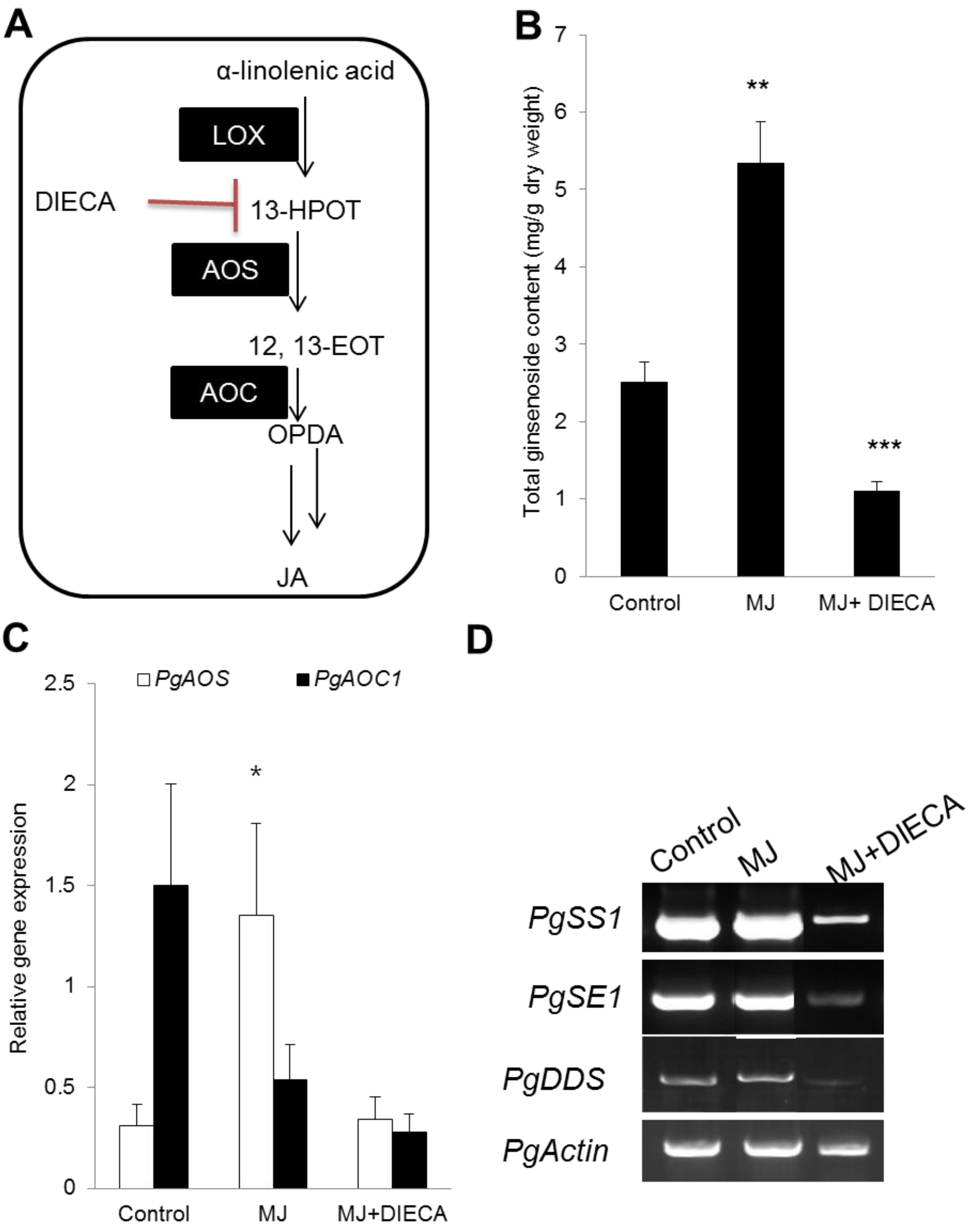

**Supplemental Figure S1.** Suppression of ginsenoside biosynthesis by JA inhibition. MJ (100 μM), an elicitor of ginsenoside production was treated to four-week-old adventitious roots and for inhibition study, 500 μM of the diethyldithio-carbamic acid (DIECA), JA pathway inhibitor was applied to MJ-treated roots after 30 min. Roots were harvested after 3 d. A, Schematic model of JA pathway inhibition by DIECA. B, Total contents of major ginsenosides (ginsenoside Rg1, Re, Rf, Rb1, Rb2, Rc, and Rd; milligrams per gram dry weight). C, Steady-state transcription levels of *PgAOS and PgAOC1* were analyzed in treated roots. The Ct value for each gene was normalized to the Ct value for β-actin and calculated relative to a calibrator using the equation $2^{-\Delta\Delta Ct}$. Data represent the mean ±SE of three independent replicates and it was statistically analyzed and compared with controls (*P<0.05; **P<0.01; ***P<0.001) using Student’s t test. D, Gene expression of the *PgSS1*, *PgSE*, *PgDDS* in treated roots. The actin was used as an internal control.

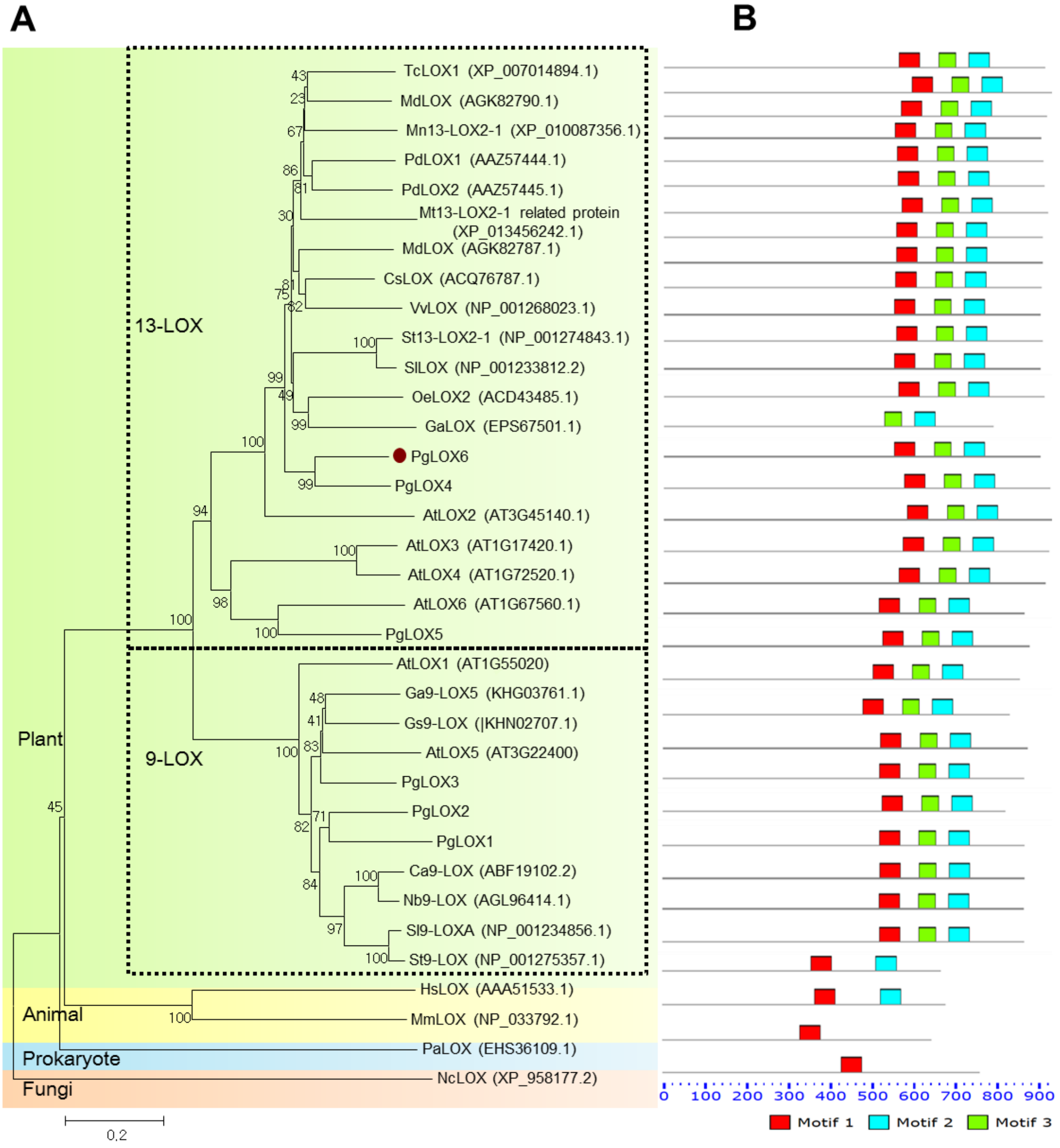


**Supplemental Figure S2.** Structural features of PgLOX6. A, A phylogenetic tree of LOXs from various organisms. The neighbor-joining method was used, and the branch lengths are proportional to the divergence, with the scale of 0.2 representing 20% changes. B, The conserved motifs among the members are highlighted in colored boxes with an arranged number, and the sequences of the motifs are depicted in Supplemental Figure S4.

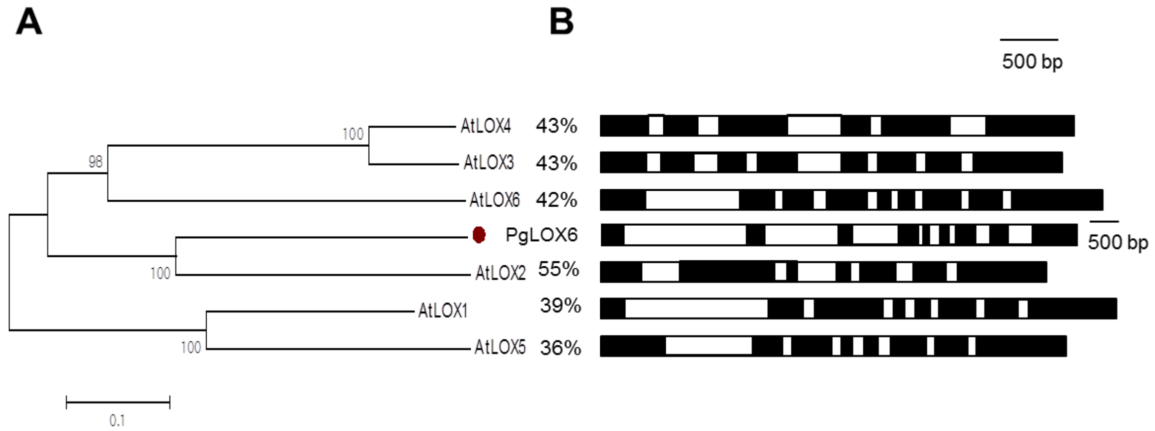


**Supplemental Figure S3.** Structural features of PgLOXa6 compared with AtLOXs. A, phylogenetic tree of PgLOX6 and LOXs from Arabidopsis including AtLOX1 (AEE33175.1), AtLOX2 (AEE77997.1), AtLOX3 (AEE29585.1), AtLOX4 (AEE35334.1), AtLOX5 (AEE76630.1), AtLOX6 (AEE34664.1). The neighbor-joining method was used, and the branch lengths are proportional to the divergence, with the scale of 0.1 representing 10% changes. B, DNA structure of *PgLOX6* and *AtLOXs*. Black and white boxes indicate exons and introns, respectively.

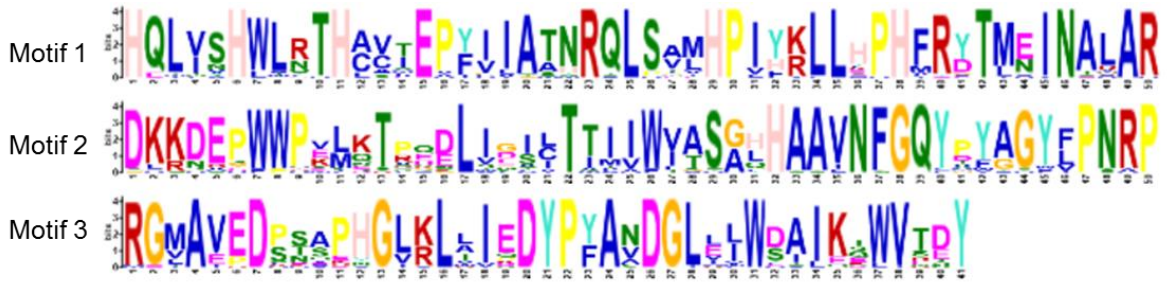


**Supplemental Figure S4.** Conserved motifs among LOXs. Conserved residues analyzed by MEME are represented by three motifs.

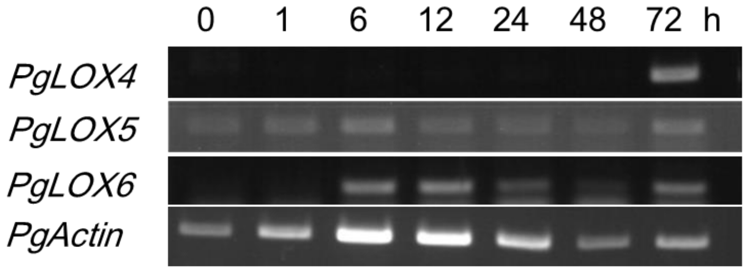


**Supplemental Figure S5.** Induction of *PgLOX6* and other 13-LOXs by MJ treatment in 1-month-old ginseng seedling. MJ (50 μM), an elicitor of ginsenoside production was treated to the one-month-old ginseng seedling. Transcription levels of PgLOX6 along with other 13-LOX genes (*PgLOX4* and *PgLOX5*) were analyzed using the same seedling.

```
ACTTATTACGCATCTCTTTAAAAAATTTACTAATAATACTTATTCATGTAACTGTCAATCAAATAATTGAGATAAACATTCCTAATATATATTATTTTTT -900
TTAATCAAATAAGGGGTGATTACTATTATTGTCAGAATATATGTTGAAGATAATTTTTTCTGAAACATTTTTGGCAGAGTGGGGCACACATAATTTAGAT -800
AATTCCCATGCATTAGTTTTTAAAATGTCATGCATAAAATTGCAATGACTAAGACATCCCAAAGTTTTTATTTTCGGTGACAGAATTCAATTTGCAATTA -700
ATTAATTACCATGTGAAAATAAACATGAAGATCTAGAATTGTCCAATTAATTATGTCCACCTTTTACTTAGATGACAACTTAATTATTTCAAAAATTAAA -600
GATACATATATTTTAACTTTTTAAATCAGTTAACAAAATTTTAAACTTCGAAATGATATGAATTTATTTGGTTTTAACAAAAACAAATATAATTATAATA -500
AATTAACTAAATTGTATAATTAATGATATATTTGTTAATGGCTTTCAAAACTTAAAACAAAATCATATCAGTTTTGAAATCAGAATTTTATAAATTAATT -400
TTAAAAATACAAATATATCATTAATTTTGAGACTTGTGTACCATTCAAATTAGGTTGGTTTTAAAACGTGTTTGTATATTATAGTGAGATATTCCCTCCA -300
TACATACACAAATGAAAATGATAGTCTTACACTCCTACTGGATTTTTAATTAATATAATTTTGTAAATATATATAAATCAAAATTAAAATGGGCATCATT -200
TTATTCATTTGGAGGGATTAACTAATATATATATGCATTCTTTTTCACGTGAAATAAAAACGTATGGCCAATATTTGAGATGAATCTAATGAACTTCTTT -100
GATTCTTCTTCTATATAAAGCGACCTTAGACCTCCACAATTCATGCACCAACCCTTGTGAACAAATCAAATACCCATAGCCAAAAACAAGTAGTAACTCA -1
ATGTTAAACTCTCAGCTTCACAAATACTCGACACCCCTCTCAACCAAAACCCTAATTCCATGGCACAAACCCTTTATCTCCGGCGAAGCTGCCGGAGCTT 100   Exon 1
 M  L  N  S  Q  L  H  K  Y  S  T  P  L  S  T  K  T  L  I  P  W  H  K  P  F  I  S  G  E  A  A  G  A
CATTTTCCAAGCATCAGGAGTCTAAGCCTAGTAAGAAAAACAAGCCCTTTCGGGTTCGTTACGTGCCCGGAAGTAGGATTAAAGCGGTGCAGGTTACACC 200
S  F  S  K  H  Q  E  S  K  P  S  K  K  N  K  P  F  R  V  R  Y  V  P  G  S  R  I  K  A  V  Q  V  T  P
TGCCACCACCGATAAGGCCACCAAGGTTGTAGCGACGGTTATTGTGCAGGTAACCGTCGGTGGATTTTTGTCGAACTTAGGGTGGAAAAGAGGGCTGGAT 300
  A  T  T  D  K  A  T  K  V  V  A  T  V  I  V  Q  V  T  V  G  G  F  L  S  N  L  G  W  K  R  G  L  D
GATATTACTGATTTGTTTGGTAAATCTTTGAAATTGGAGCTTGTTGCTGCAGAGCTTGATCCTGGTGAGTATTTTTGTACAAATAGATATATATATATAT 400
 D  I  T  D  L  F  G  K  S  L  K  L  E  L  V  A  A  E  L  D  P
ATATATGTAGTCTTTAGATTCTTGAACCTATTTTTTCTTTTTTTTAGTTGGTGTCTTTCATACTCGGTCATCAGAAAAAATATTATTGGGTGAGTCACTA 500
GTTCAATCAGTAGTTGAACTCTTATTAAATCGGTGACATCATAAATTTATAAATATATATTATTAGTAATTAATAAAAAATTTAAAATCTCATTTTTTTA 600
TGAATTAAAACATAATAAATTAGTTGATTTACAATATTATTAAAAGTGATTAATTAATATTAAAAAAAATTGTACAAAAAATAATAAAAAAACTATTTCA 700
CCAGATCAACTTAACCGGTTTTACCGGTTGAACCACTGCTTCGACCGATAATCAACCAGTTCAATCCGAATTTTGAACAAACTTGTTCAACTGCACCAAA 800
TTAGACCGGACCTAGAACCGGTTCCAGGTTCAACCGATTGAACCGGCTGATGTGTCTGAAAACAGTGGGTGTCAAGAACACCGGTCGCAAGTTACTACTG 900
AAAAACTAAATTTAATATCGTGTGAGCCATTAGCACACGTGGAGGTGTTTAATTTCTTTTATATGTATTTATATTTTTTATAAAATTTAAATTTTGGAAA 1000
TTCATGAATTAATGAATTTCTAAAATTTAAATTTTAATGAACATAAAAGAAAAGAAATAACAAAATTTTGGCTACTTTTCTTGTTAAATTTTTTTTGTCT 1100
AAAAATGAATTGATCTGGACGAAAAATTAAATAACATACTACAGTAAAACCTCTATTTAGGAATATTCTATATAAGAATAACCTCTACAATATTATAAAA 1200
AAAAATTGGTCCCAATTTGGGCCTATATATATATATATATATATATATATATATATATGAGAGAGAGAGAGACAATATTAATTTTAAAAGGCTACATT 1300
TTTTATAAAATACTACATTTCCTAAAAAGAACTATATTTTTCTATAAAAGACTACATTTCTTATAAAGCTCTAAACATAGCTTGAAAAGTTAGAAATGTT 1400
ATGAATTCAACAAGAAAGTGTTTATGCTAATGAGCTATTACATACCCGCAAGTTCAAAGATGTGGTAACAACTCTAAAGAGTAAATCGTTGTGCCAAACA 1500
ACTTTGCATAAAATATTTTTTGAATAATAATTATTAGAGAGTTCTTATTGATATTTTTGTTAAAGTGGATATTTTTGGGTGTTCGAAAAATATTTTGTAC 1600
CAATACTTCTATTTTGTAATAACCTCTCAATTAAAATACAAATAATTTAGTCCCAAGTCTATTCTTAAATAGAGGTTTTACTGTATCATCTATATTTTAT 1700
AATCCATATTATACTCTCTAGTTTATAGACATAATAAAACGAATTTAAAGGTAAGTTAGCAACTTAAATGTGGAACTTAAATGTTTTTTTTAATTAAACA 1800
AAATAACAACAAGAAAGAAACTTACTAATTTTTATTTTATTCTTTTCTTTTCTGCCTTTCTGGCCAATCCTCATAAAGAGTGTTAGGCTGAGGTAATTAA 1900
GAAGGGTACCATATATATTATATATACATAGTGCTAAAAACTTAATTGCAGCATAGAAACTAGAAAAGTATATATTTGACCTAATTTTTTGTGTGTCAAA 2000
TAAAAGAAATTCTTCAATATGAAATTAATTAGGGACCAAGAAACTTACAACCTTATCCTAAGAAGAACATGCATGCATGATCCTATTATTGGATCTAAAA 2100
AGTCCTTAAAACCTTGGCCTACGAAGTAGAACTACTGCGAAAGTCCAAGAGGAATTAACATCTGTTGTCTCTTTCTTTTTCTTTTAGTCCCCCACCAATC 2200
TTACAAAAATCGCTTTTGTGAGGTTATATCAAGAATCTCATTTCATTTGTGTCCTTGCAAAATTGGTTTTATTACAATTCCGCCCTTGTTATTTTTTATT 2300
TTTTTTATTCTACTCCAAAATTTGATAAAACTTCTATTTATATATCTTCTTTACTATAATTACACACGCAACGCGTGTACCACCGGGACTAGATTATTTA 2400
ATGTTATTCTTCTTCTTTTTCCTCATTCAGTATTCTTGGCTTCTTTTTTTTTTTTAAAGAAAATATCTAACAAATCTTAAATTTGTTTCATAAGCTACCG 2500   Exon 2
                                                                                                   A  T
GATCAGAGAGGGAAACAATCCCAGCGTTTGCATCCCGGGCGGGGCGAGAAGACGTGGATGACATAAAATATGAGTGCAAATTTACAATTCCACAAAATTTT 2600
G  S  E  R  E  T  I  P  A  F  A  S  R  A  G  R  E  D  V  D  D  I  K  Y  E  C  K  F  T  I  P  Q  N  F
GGGGAGATTGGAGCAATTTTGGTAGAAAATGAGCACCGCAAAGAAATGTTCCTCAAGCATATTGAACTTGATGGATTCCCTAACGGTAGAATTAATATTAAT 2700
  G  E  I  G  A  I  L  V  E  N  E  H  R  K  E  M  F  L  K  H  I  E  L  D  G  F  P  N  G  R  I  N  I  N
TGTGAATCATGGGTTCATTCCAAGTACGATAATCCTGAGAAGAGAATTTTCTTTACAAGCAAGGTCAGTTAATTACTCCATCCGTGCTCACGTTTTAGAGT 2800
 C  E  S  W  V  H  S  K  Y  D  N  P  E  K  R  I  F  F  T  S  K
TGTGTACAAAAATTAAAGAAATAATTATATAACATTAATGCAATGTTTGGATTGGAGTAATGAAGGTGAGAATGGAATGACTCATTCCCATAAAATGAAAA 2900
AAAATTATATTTGGTTGCAATTGGAATGGATTGAATCATTCTTAATTATTCCTCCCAAATTTATGAATTTTATTCCTTGAGTGATGAAGAATGTGAAATAA 3000
CTCATTCTAACTAGAATGGAATGATCCATTCCATTACTTCAATCCAAACGTATCATAAATATAGTATTAATTATATATAATACTATTTACCATATATTTT 3100
ATCGATCTCATATTTAACATTTATCAATAAGGGTAATTTTGTAAAAATTATAATTAATTCTTTTAAATTATATAAAATATTAAATAATTTAAAAAAATAA 3200
AATTATCCAAAACGCCATATATTATGAAAAATAACCCAATTTTTGATCGTCTTTTCCGGTTAATTGAAAAGCTCATAAAAAACAAATAGAAAACATTTTT 3300
ACGTCCTTTTCTTTATTGCGGTCCTTTTACTAAGATATTAGTTGTAGGAGGGAATCTCGTACTATTTTTGTCGTGATATAATAATGATATTAATGTAATA 3400
ATGTAACATCTTAATATGTGAAATTATATGTAATAATGTAACATCTTAACATAAATGTATACAAAACAAATAATTGGTTAATAATATTTTATTATGTTAG 3500
TACTAATTAATTTGTTAATTAAAATTACGTTACTTAGAGATCATTATGTACTATGTCAGTCTGACATGTATAGTTTTTCATATAGTTATGCAACAAGACA 3600
AATTTTTGTTTCATAATTAAGCTAAAATTGAATAGGACAACTCAAGATTTATTTCGCCAAACTCTAAAAATAATGGAGTCAATTTTTATTGTTATTATTG 3700
AAAAGGATTTTTTTTTTACTATAATTGTATTTAAAAAATAATTCAGCTCTAGTTATAAATAAAAACTGACATTAACCTCATAATTATAGAAATTTAACCT 3800
TGCCTTTTCTCTAGAATGTTTTATTCTTACCGAAAATGTGACTTATATGAGCCATATTTCACTTGTGATTATTGTAATTACGAAAGAAATAATTGATATT 3900
AATTATAATCTACAACCGTTTCAAAAAAGAAAAGAGGAAATTTATAATGTCTAATTTTTTTAATTTCTTATTATTATTATTTTTTTGGTAATTAACTTGT 4000
```

**Exon 3**

GATTTATTGAATGACAGTCATTCATACCCTCTAAAACACCAAGTGGGCTAGTAAAATACAGAGAAAATGAACTGCTAGCACAGAGAGGCGACGGCTCCGGC 4100
S F I P S K T P S G L V K Y R E N E L L A Q R G D G S G

GAGCGGAAGGAGAGTGACAGAATCTACGATTACGATGTCTATAACGATATCGGCGATCCCGATAAAAATAAAGATTTGGCCCGACCAGTTCTCGGCGGCAAG 4200
E R K E S D R I Y D Y D V Y N D I G D P D K N K D L A R P V L G G K

GAACATCCATACCCTAGGCGTTGCAGAACTGGCCGCTCCCGCACCAAGGAAGGTAATAAATTTTAGTCACAAATTTGTTACCCTTTTCAAGATTTCATATGC 4300
E H P Y P R R C R T G R S R T K E

TTGATCATATTGATCATACTCATCAAATATCTGTAAATGGGGTTGCAAACCGAATTGGGTTTGCTTGATTTCAAGCAAAAGCTACTTCTTTTGCTTGAAGT 4400

AGTCCCATTTATTTGTGTGGTTGACACACTTTTATGTTTTATTAAGAAAATTAATTTTTCCCCCTTCACAAATGTTGGTAGGTTTGGGACTTTAAAGCACC 4500

TAAAAGATTCAAAAAGTTTAACGGGTAGTATGTTTTTTAACCATTAAAACTTTAGTACAATTTTTAGTGAAAAGATGGTAAAGCGTCGCCATAGATATTCT 4600

GGTCTATGCCGACGGTTGAATCGTCCCTATAACTCAAAAAAATCGTCGTCGCACTGTGTATATACATATAACTCATATATATATATATAGGGTTCCATCCA 4700

.CAGCAACCGGTTGCTGTGGAGCAACC( TTTTTTGGCCTGTGAAATTTCGGCTGGAGCAAAAAAAATAAAAATTCGCAGGCCAAAAGGGGTTGCTCCACAGC 4800

AACCGGTTGCTAAAGATGAAACCCCTATATATATTTATATATATATAAACATATTTTAATGATAACTAGAACTATTTGATTTTGGAATAAACCGAAGGTTT. 4900

AAACATATTTTAATGATAACTAGAACTATTTGATTTTGGAATAAACCGAAGGTTT.AGATCATCAATATGATATACTACATTGTATTATCTGCAAAATAGGC 5000

ATAGTGCAAAATATTAGTACTAGTTTTTGACGACTATGTCGTTTGTGCTGTAAATAATATACATGCATATACTTTGCGTATACAGACCCATTGTCAGAA 5100
D P L S E

**Exon 4**

GAATCAAGGAGCAGCTTCGTTTACGTGCCAAGGGATGAAACTTTCTCGGAGGTAAAGGGGCTAACATTCTCGGCAAAGACAATATACT 5200
E S R S S F V Y V P R D E T F S E V K G L T F S A K T T Y

TCGGTGATTCATGCAGTTCTACCTTCGCTAGAAAATGCCGCCATCGACAGTGGACTTTGGGATTTCCATACTTCACAGCCATTGACTCACTGTTTCAATGA 5300
S V I H A V L P S L E N A A I D S G L W D F H T S Q P L T H C F N E

AGGGGTTGATCTGGGAGAAGAACCTAAAGCCACACTTTTCAATCTTCTTCCTAGACTTGTCAAAACTATTACTGATCAAGGAAAAAACGTCCTACTTCCCT 5400
G V D L G E E P K A T L F N L L P R L V K T I T D Q G K N V L L A L

AGACTCCTGAATTGCTTGACAGGTACGAACAAAATCTCTCTCTATATATGTGCGTCTTTCTAGGCCTATTGCTTGTATTTCACCATTTTCGTGTATATTGA 5500
E T P E L L D R

**Exon 5**

TCTGCAGGGATAAATTTGCTTGGATGAAGGATGAGGAATTTGGTCGTCAAACCTTAGCTGGTCTTAACCCTTATAGCTTAAGATTGGTTACAGAIATGTTC 5600
D K F A W M K D E E F G R Q T L A G L N P Y S L R L V T E

TACCAACACTTCTCAGAAATGCAGAAGCATTTTTTTTATTAGAAAAAAATAAAAATCTAAAGTGTATGATTTTGGCACATTCAAGTAAACTCCTAGAAATG 5700

**Exon 6**

GTCTAAAAGTTCTTCTATATATGAATTTAGAAGCAACAGTTCTAGAAAAATTGCTTTAATTAATTATTCTTATGCGCAGGATGGCCATTGAAAAGTGTTTG 5800
W P L K S F

TGACCCTGGGACGTACGGACCAGCTGAATCAGCAATCACCGAAGAGTTGGTCGAACAACAAATCAAAGGCATCATGACAATTGAGGAGGTAATAAGAATAT 5900
D P G T Y G P A E S A I T E E L V E Q Q I K G I M T I E E

ATCCCACCTAGAAACATATGATCGTATTCATCTTTAATTCTTCCCAAAAAAAAAGAACAACAGAATTGTTTTGTAACTCGATTGAATTTGTATATAAATGC 6000

**Exon 7**

AGGCCATTAAACAAAAGAAGCTTTTTATTCTCGATTACCATGATTTGCTCTTACCGTATGTGAACAAAGTAAGAGAACTAAAAGGGACAATTCTCTATGGA 6100
A I K Q K K L F I L D Y H D L L L P Y V N K V R E L K G T I L Y G

TCAAGGAC TTGTTTTTCCTAACTCCTATTAACACATTAAAGCCAGTAGCCATTGAGCTAGTTCGGCCACCGGGAAAAGGAAAACCCCAGTGGAAGCAAGC 6200
S R T L F F L T P I N T L K P V A I E L V R P P G K G K P O W K O A

ATATAGG( CCGGTTGGGATGCTACATCTGCCTGGCTTTGGAAGCTAGCAAAAGCTCATGTCCTGGCACATGACTCTGGTTATCACCAGTTAGTTAGTCATT 6300
Y R P G W D A T S A W L W K L A K A H V L A H D S G Y H Q L V S H

GGTAGGTTCCATTTAGACATCTCTCTGTGTGTCATTTATGTAATTATGAGGAGTTATATATATTATAAAGGACGAGAGGGGGTGGGGGTGGTCATTTTTT 6400
W

TATCCCTATTTTCTATCATATCTTTTCTCATCCATACTTTTTAAGTTTTATTTAAATATTTTATTTTTATTTAATTACACATACTATGTGTGGTGTGTAC 6500

**Exon 8**

CACATAAACTAGCGAGAAATATGCCGATATCTTTTCTCAATTCTGTCTCTATGTTTTGTACTTTGCAGGCTCWAAGAACTCATTGTGCCACAGAGCCATAT 6600
X R T H C A T E P Y

ATAATTGCGACCAACCGACAACTAAGTGCAATTCACCCAATTTATAGACTATTACACCCTCATTTTCGATACACAATGGAGATCAACGCTCTAGCGCGAC 6700
I I A T N R Q L S A I H P I Y R L L H P H F R Y T M E I N A L A R

AAGCTCTTATTAATGCAGGCGGAATCATTGAGTCATTGTTCTCACCTGGCAAATACTCTATAGAGTTTAGCTCCGTCGCTTATGACAAGCAGTGGCGCTT 6800
Q A L I N A G G I I E S L F S P G K Y S I E F S S V A Y D K Q W R F

'TGATCAGGAAGGATTGCCAGCAGATCTCATTAG AGGTAATAAGTTAATCCAAAGGCTTAACAAAGAAACTAAACTAAAAATTCAACCAATCACATGCCA 6900
' D Q E G L P A D L I S

TTTTAACAAAAACATTTGCTTTGCTACCAAGTACTTAATGGCAAAAAAAATATTACTTTACCCTATTTATTTGGTAGTATTACATTCGTGTCACTGAAAT 7000

AAATAGAGAAGGTATGCTGAAGTTTTGTGAAAAATAAATTTTTGTCTTACATTTGCTATAACCTTTCAAACCAGAAGAGGCCTTTATCTTTATCTTTTTTC 7100

ATCAAATATTTATAAGAACAATTGGCCATAAGGTACTTGGTCGATCTATAAGTTTCTGTGGAAGTACATATATAGTCTAATAGTGATTGACTAAAGGAAA 7200

**Exon 9**

.TTATAAAATAAGCAACT CATACAATTTTCTTCATTGTAAATTTCTAGGGGAATGGCCGAGGAAGATCCAACAGTACCGCATGGCCTTAAGTTGGCCATA 7300
R G M A E E D P T V P H G L K L A I

GAAGACTACCCTTTCGCTAACGATGGTCTAATCCTTTGGGATATCATTAAAATGTGGGTTACAGACTACGTCAGCCATTACTACCC( GAATCAAGCCTGG 7400
E D Y P F A N D G L I L W D I I K M W V T D Y V S H Y Y P E S S L

GTAGAATCTGATGAAGAACTTAATGCATGGTGGAAAGAAATCCGAACAGAAGGCCATGCAGACAAAAAGGATGAACCGTGGTGGCCTGTTCTCAAAACCC 7500
V E S D E E L N A W W K E I R T E G H A D K K D E P W W P V L K T

CTCAAGACCTAATCGGAATCCTCACAACCATCATTTGGGTAACATCTGGCCACCATGCGGCGGTTAACTTCGGGCAATATCATTACGCAGGATATTTCC 7600
P Q D L I G I L T T I I W V T S G H H A A V N F G Q Y H Y A G Y F

CAAACAGACCTACAATCGCAAGAACCAAAATGCCCACAGAAGATCCAACCGAGGAATCTTGGAAATCTTTTGAATTAAGACCCGAGGATGAGATCCTATC 7700
P N R P T I A R T K M P T E D P T E E S W K S F E L R P E D E I L S

GTGCTTCCCTACTCAAATTCAAGCTACTAGAGTTATGGCAATTTTGGACGTGTTGTCGAATCATTCGCCCGATGAGGAATATATTGGAGCGGAACCAGAG 7800
C F P T Q I Q A T R V M A I L D V L S N H S P D E E Y I G A E P E

CCAGCATGGGCAGACGAGCCAATTATAAACGCAGCATTTGAAGTTTTTAGTGGAAGGCTGAAGGAGCTTGAAGGGATTATAGATGCAAAAAATGCAGACA 7900
P A W A D E P I I N A A F E V F S G R L K E L E G I I D A K N A D

AAAACTTGAGAAATAGAACCGGAGCTGGGGTTGTGCCGTATGAGCTTTTAAAGCCATATTCCAAACCTGGAGTTACAGGGCAGGGAGTTCCTAATAGCAT 8000
K N L R N R T G A G V V P Y E L L K P Y S K P G V T G Q G V P N S I

TTCCATTTGAATCAGACATTTGAGGAATTAAGGGCAAGCTTTAATATTATCACTTGGTGTTGCAATATAGACTAAATAAATTACTTTATGGAACTACTGA 8100
S I *

**Supplemental Figure S6.** Genomic DNA sequence of *PgLOX6* with its promoter (–1317 to –1), and a deduced amino acid sequence. A one-letter code below the corresponding codons and intron sequence is shown. Square boxes in the intron region: putative intron-exon boundaries with the GT-AG rule.

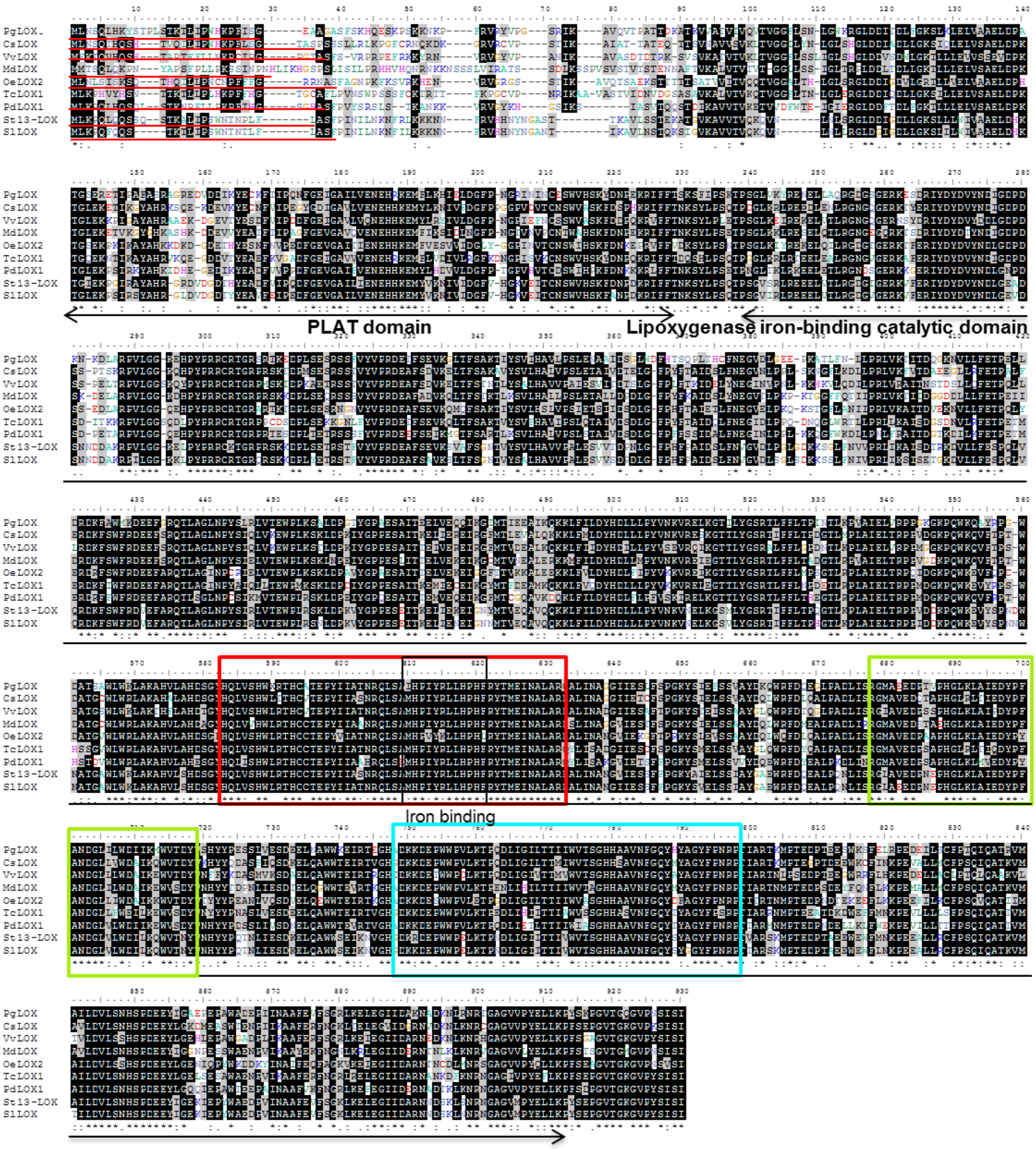


**Supplemental Figure S7.** Multiple alignment of the deduced amino acid sequences of PgLOX6 with homologous LOXs from other plants; *Camellia sinensis* (CsLOX, ACQ76787.1), *Vitis vinifera* (VvLOX, NP_001268023.1), *Malus domestica* (MdLOX, AGK82787.1), *Olea europaea* (OeLOX2, ACD43485.1), *Theobroma cacao* (TcLOX1, XP_007014885.1), *Populus deltoides* (PdLOX1, AAZ57444.1), *Solanum tuberosum* (St13-LOX, NP_001274843.1), and *Solanum lycopersicum* (SlLOX, NP_001233812.2). Black

boxes indicate identical residues; gray boxes indicate identical residues for at least two of the sequences. Red underlined sequence showed the proposed signal peptide. Conserved domains are indicated with arrowed lines and three conserved motifs are highlighted in colored boxes. Iron binding is indicated with a thin square box.

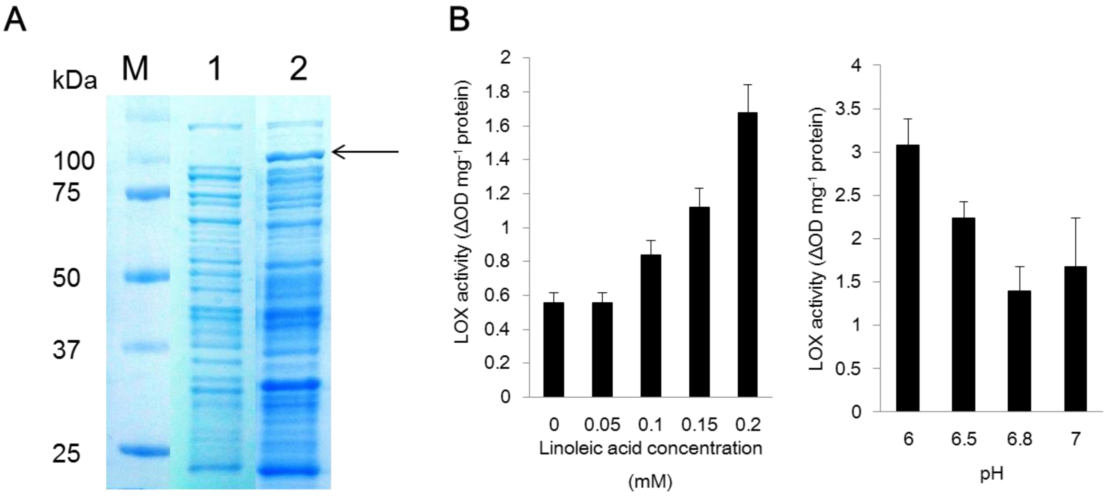


**Supplemental Figure S8.** Biochemical assay of PgLOX6. A, M = molecular mass standards are indicated in kDa; lane 1 = control (*E. coli* transformed with pMAL-c5x carrying PgLOX6); lane 2 = total protein extract after 8 hours of 1 mM IPTG induction of *E. coli* transformed with pMAL-c5x carrying PgLOX6. B, Extract of IPTG-induced *E. coli* transformed with pMAL-c5x carrying PgLOX6 was used to find the optimal function of LOX at different concentrations of substrate linoleic acid (mM) and at different pH.

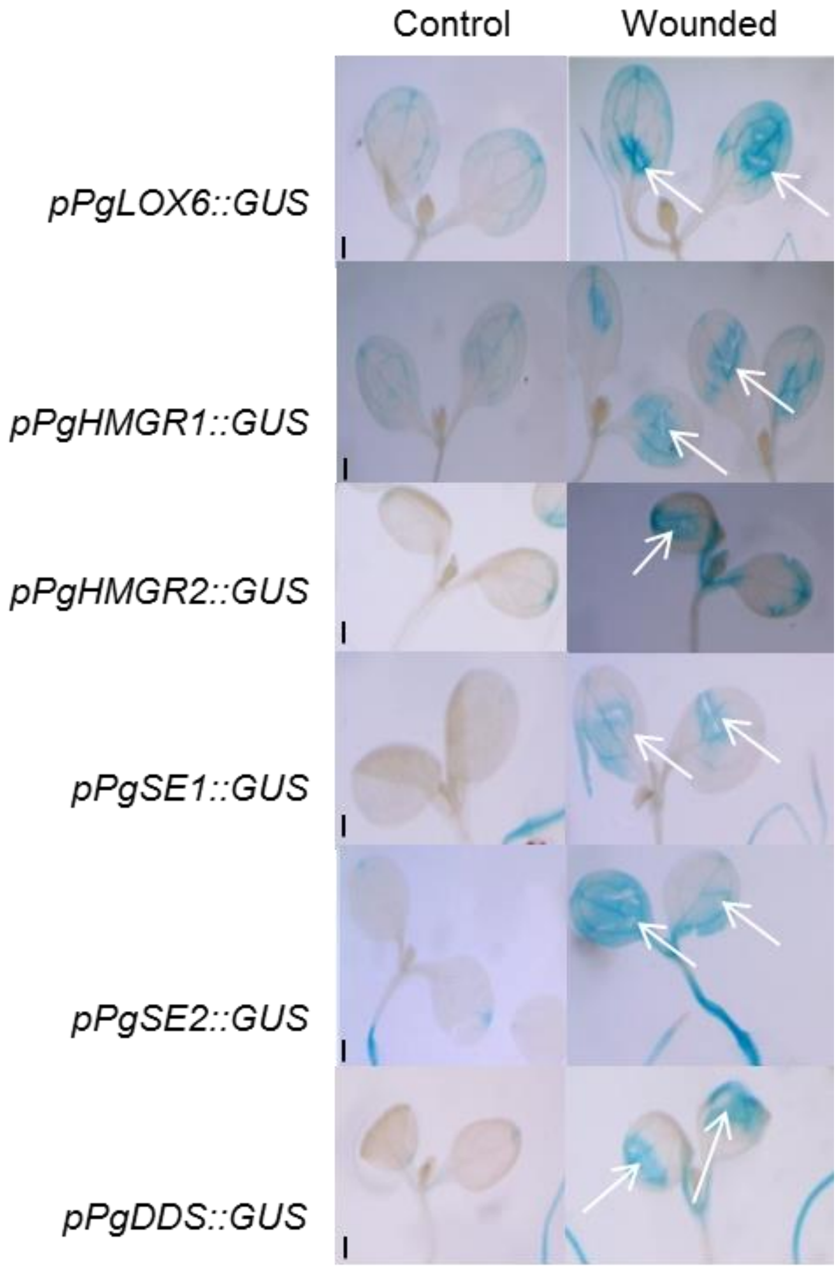


**Supplemental Figure S9.** Effects of wounding on *pPgLOX6::GUS*, *pPgHMGR1::GUS*, *pPgHMGR2::GUS*, *pPgSE1::GUS*, *pPgSE2::GUS*, and *pPgDDS::GUS*. The plants were grown in ½ MS solid medium for one week. The seedlings leaves were pricked with a needle and harvested after 24 h for GUS histochemical analysis. Bars = 100 μm.

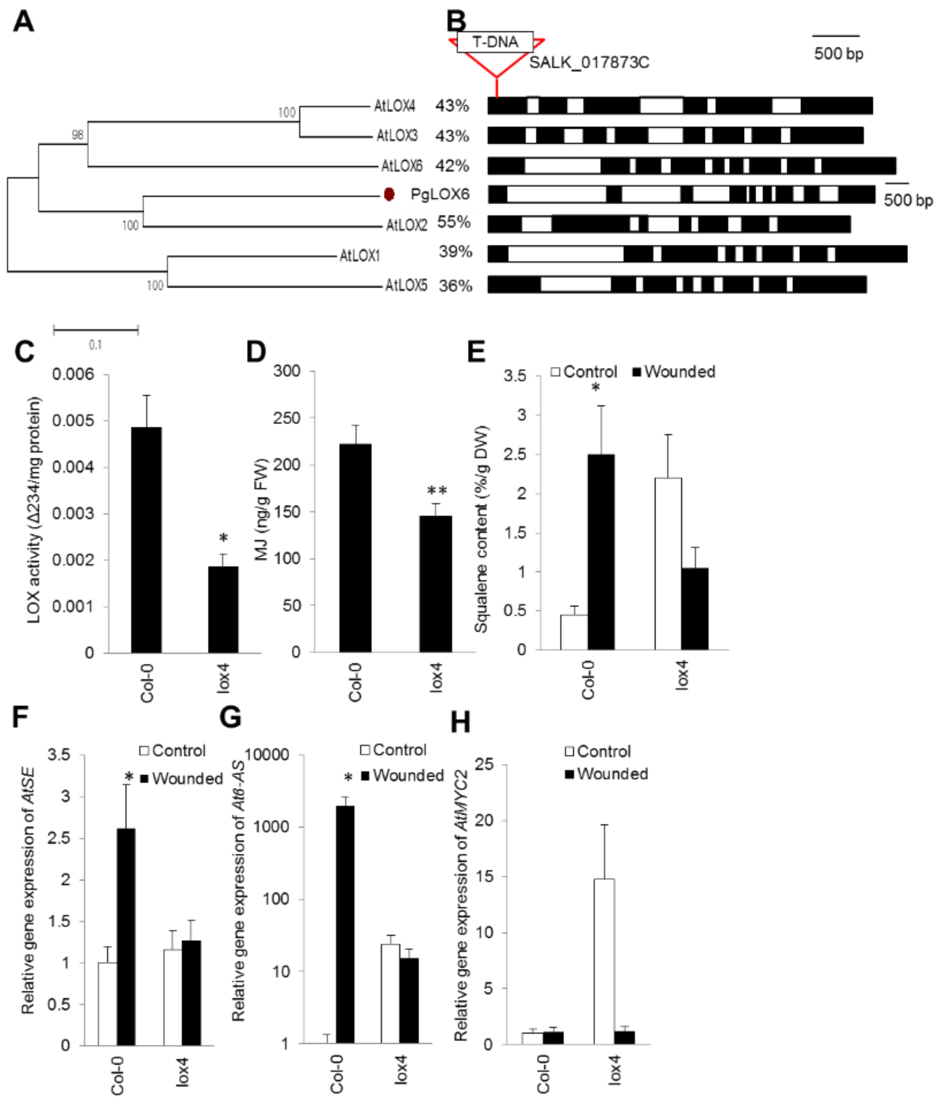


**Supplemental Figure S10.** Lack of LOX-derived JA decreased triterpene content in wounded Arabidopsis. A, phylogenetic tree of PgLOX6 and LOXs from Arabidopsis including AtLOX1 (AEE33175.1), AtLOX2 (AEE77997.1), AtLOX3 (AEE29585.1), AtLOX4 (AEE35334.1), AtLOX5 (AEE76630.1), AtLOX6 (AEE34664.1). The neighbor-joining method was used, and the branch lengths are proportional to the divergence, with the scale of 0.1 representing 10% changes. B, DNA structure of PgLOX6 and AtLOXs. Black and white boxes indicate exons and introns, respectively. Structure of AtLOX4 transfer DNA (T-DNA) insertion position (SALK_017873C). Arabidopsis *lox4* mutant and Col-0 were grown for five weeks and pricked with a needle. The wounded and control leaves were harvested after 3 days and used for analysis. C, Activity of LOX in Arabidopsis *lox4* mutant and Col-0. D, Quantification of endogenous MJ level in Arabidopsis *lox4* mutant and Col-0

using LC-MS. E, Quantification of endogenous squalene in wounded and control Arabidopsis *lox4* mutant and Col-0 using GC-MS. F, G, and H, Steady-state transcription level of *AtSE*, *Atβ-AS*, and *AtMYC2* in wounded and control Arabidopsis *lox4* mutant and Col-0. The Ct value for each gene was normalized to the Ct value for β-actin and calculated relative to a calibrator using the equation $2^{-\Delta\Delta Ct}$. Data represent the mean ±SE of three independent replicates and it was statistically analyzed and compared with controls (*P<0.05; **P<0.01; ***P<0.001) using Student's t test.

**Supplemental Table 1.** Primers used in this study.

| No. | Plant | Gene | Primers sequence (5'-3') |
|---|---|---|---|
| 1 | Ginseng | *PgLOX6* | TATACCCGGCTGGTTTTCTG<br>ACTTTGTTCAATTCTAAATCCGAA |
| 2 | Ginseng | *PgAOS* | AAAATACCAGTCGACTGTTTTCCGAG<br>TGAGTTCAGTGGAGGGCATAAATGT |
| 3 | Ginseng | *PgAOC1* | CGGCGATCTTGTCCCTTT<br>TTGCTGAAGCTTAACCTGACC |
| 4 | Ginseng | *PgHMGR1* | CCAGTTTTTAAAGCAAACACTAGG<br>GGTTGGAGACGGCGAGCGATCTAC |
| 5 | Ginseng | *PgSS1* | ATGGGAAGTTTGGGGGCAATTCT<br>GTTCTCACTGTTTGTTCAGTAGTAGGTT |
| 6 | Ginseng | *PgSE1* | AGCAGCAGTTGACAAAGG<br>GCCACATTCGTTTTGGTGAAGG |
| 7 | Ginseng | *PgDDS* | ATGTGGAAGCTGAAGGTTGCTCAAGGA<br>TTAAATTTTGAGCTGCTGGTGCTTAGGC |
| 8 | Ginseng | *PgAP2* | AAACCCGGGTAAGAAAGGTG<br>GAGCCAATCAAATTC |
| 9 | Ginseng | *PgWRKY1* | CAAGCGAAAGTGCAGTTCAA<br>CTGGGCAACCCCTTACACTA |
| 10 | Ginseng | *PgWRKY22* | CCCACAAAGAAATCGTCGTT<br>GGTACAAACCGGCACAAGTT |
| 11 | Ginseng | *PgDELLA* | CGGTGTTTTTGGACAGGTTT<br>TGAGTCAAGGTCTCGTGTCG |
| 12 | Ginseng | *PgERF3* | TCATCAGCGAGACCAGTGAC<br>CAGATGGCTGCTTCCTTTTC |
| 13 | Ginseng | *Pgβ-actin* | GTGATCTTACAGATAGCTTGATGA<br>AGAGAAGCTAAGATTGATCCTCC |
| 14 | Arabidopsis | *AtAOS* | GCTTTTATCGCCGAGAATCCA<br>TTGAAACTCAGGGAAGATCCGGT |
| 15 | Arabidopsis | *AtMYC2* | AAGAAAGCCAGCAAACGGTAGAGA |

| | | | |
|---|---|---|---|
| | | | CTCGAGCTGGTTCTTGATTTGGA |
| 16 | Arabidopsis | *AtSS1* | AGGCGATTTCCAATTCAATG |
| | | | AAGACCTCGCCTCAGTTTCA |
| 17 | Arabidopsis | *AtSE1* | AGCTGGTGTTGCTGGTTCT |
| | | | CTTCCACACAATCTTCAATTCC |
| 18 | Arabidopsis | *Atβ-AS* | TAACGACTGTGAGGCAATACGC |
| | | | TCATGGCAACGAAACTCGCGC |
| 19 | Arabidopsis | *Atβ-actin* | GTGTGTCTTGTCTTATCTGGTTCG |
| | | | AATAGCTGCATTGTCACCCGATACT |
| 20 | Vector | *CFP, GFP* | CTCGACCAGGATGGGCAC |
| 21 | | *35S promoter* | GCACAATCCCACTATCCTTCG |
| 22 | | *NOS terminator* | ACCGGCAACAGGATTCAATCT |

**Supplemental Table 2.** *PgLOX6* contributes to JA production in MJ-treated transgenic roots. JA and MJ contents were quantified by LC-MS analysis in transgenic ginseng roots expressing *PgLOX6* 24 h after treatment by MJ (100 μM).

| | | JA | MJ |
|---|---|---|---|
| Non-treated | Control | 19.02±0.01 | 20.9±0.005 |
| | 35S:LOX6#8 | 18.95±0.007 | 20.82±0.03 |
| MJ-treated | Control | 191.43±21.27 | 790.61±87.85 |
| | 35S:LOX6#8 | 235.06±26.12 | 2671.72±178.11 |
